# Synthesis and characterization of water-soluble C_60_–peptide conjugates

**DOI:** 10.3762/bjoc.20.71

**Published:** 2024-04-12

**Authors:** Yue Ma, Lorenzo Persi, Yoko Yamakoshi

**Affiliations:** 1 Department of Chemistry and Applied Biosciences, ETH Zürich, Vladimir-Prelog-Weg 3, 8093 Zürich, Switzerland

**Keywords:** biomaterial, fullerene, peptide, water-soluble

## Abstract

With the aim of developing biocompatible and water-soluble C_60_ derivatives, three types of C_60_–peptide conjugates consisting of hydrophilic oligopeptide anchors (oligo-Lys, oligo-Glu, and oligo-Arg) were synthesized. A previously reported Prato reaction adduct of a biscarboxylic acid-substituted C_60_ derivative was subjected to a solid phase synthesis for amide formation with N*-*terminal amines of peptides on resin to successfully provide C_60_–peptide conjugates with one C_60_ and two peptide anchors as water-soluble moieties. Among three C_60_–peptide conjugates prepared, C_60_–oligo-Lys was soluble in water at neutral pH, and C_60_–oligo-Glu was soluble in buffer with a higher pH value, but C_60_–oligo-Arg was insoluble in water and most other solvents. C_60_–oligo-Lys and C_60_–oligo-Glu were characterized by ^1^H and ^13^C NMR. Photoinduced ^1^O_2_ generation was observed in the most soluble C_60_–oligo-Lys conjugate under visible light irradiation (527 nm) to show the potential of this highly water-soluble molecule in biological systems, for example, as a photosensitizer in photodynamic therapy.

## Introduction

Since the seminal discovery in 1985 by Kroto, Smalley, Curl, and co-workers [1], fullerenes, specifically buckminsterfullerene C_60_, have intrigued the scientific community. The unique structure of fullerenes, characterized by a fully conjugated closed-cage structure, containing a mixture of hexagonal and pentagonal rings, have been recognized for the unique electronic [2–4], optical [5–6], and mechanical properties [7–8]. Despite the notable achievements in fullerene research and the potential applications in diverse fields, a significant obstacle remained for the use in biological studies: fullerenes are poorly soluble in polar solvents, including water and other water-miscible solvents [9]. This challenge consequently restricted the studies of fullerenes as biomaterials since related in vitro bioassay systems require water solubility of the chemicals for testing. To overcome this important obstacle, over the past decades, a variety of water-soluble fullerenes have been reported [10].

General approaches towards enhancing the water solubility of fullerenes involve either 1) covalent functionalization of the fullerene surface with polar moieties or 2) complexation with water-soluble host molecules or polymers. Related to the former approach, the Nakamura group [11], Wudl group [12], and Hirsch group [13] reported initial work in the early 1990s on water-soluble C_60_ derivatives by covalently attaching water-soluble moieties to the fullerene core. Subsequently, Nakamura and co-workers further developed reactions between C_60_ and organocopper reagents, enabling the sequential addition of functional groups to obtain penta- and decaadducts, which largely enhanced the water solubility [14–15]. As early examples of the latter approach in the 1990s, Wennerström and co-workers reported the study of supramolecular BiCAP complexation of C_60_ with γ-cyclodextrin (γ-CD) [16]. Shinkai and co-workers synthesized water-soluble calixarene derivatives to form water-soluble complexes with C_60_ [17–18]. By either chemical functionalization or complexation of the fullerene core, a number of biocompatible fullerene materials with interesting biological activities were recently prepared and reported [19–24].

**Figure 1:**
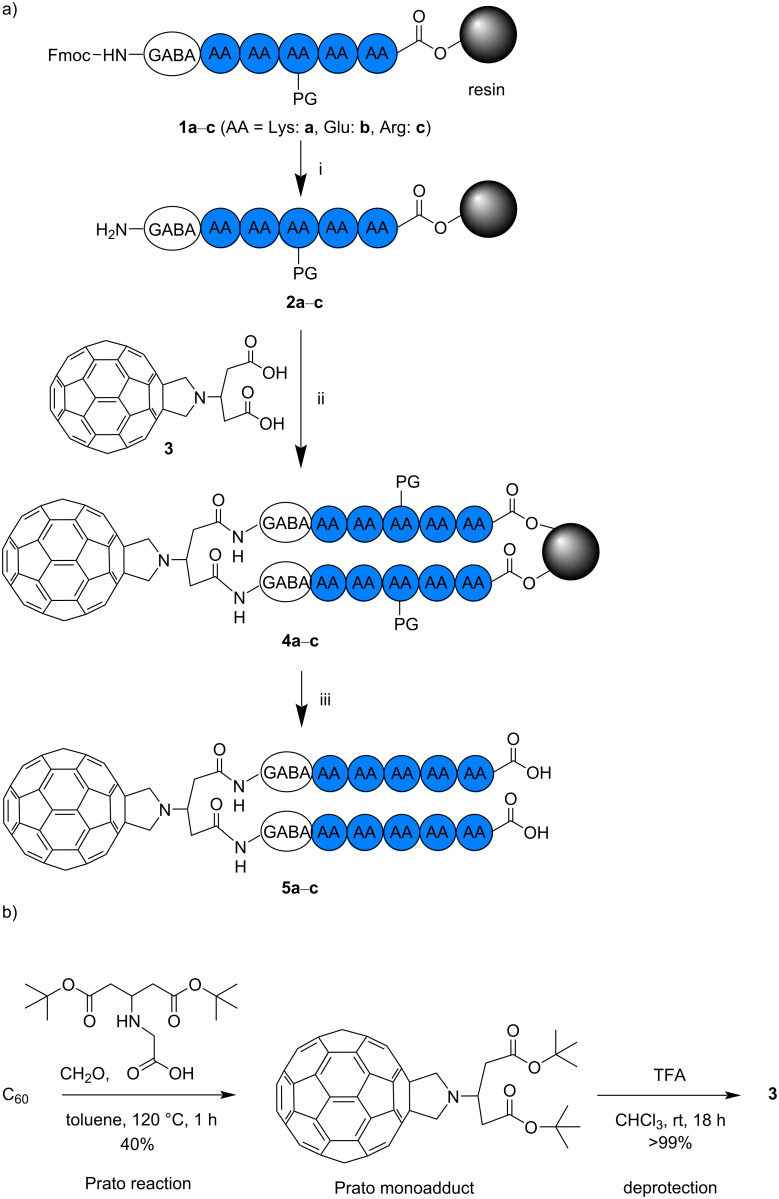
a) Synthesis of C_60_–oligopeptide conjugates **5a**–**c** and b) synthesis of compound **3**. Fulleropyrrolidine-based biscarboxylic acid derivative **3** was prepared by Prato reaction and subsequent deprotection. Compound **3** was subjected to SPPS with the peptides on trityl resin (i.e., **2a**–**c**) to provide **4a**–**c**. By simultaneous deprotection of peptide side chains and cleavage from resin, C_60_–oligo-Lys (**5a**), C_60_–oligo-Glu (**5b**), and C_60_–oligo-Arg (**5c**) were obtained. Reagents and conditions: i) 20% piperidine, rt, 2 × 10 min, ii) HBTU, DIPEA, in DMF, rt, overnight, and iii) trifluoroacetic acid (TFA)/triisopropylsilane (TIPS)/H_2_O, rt, 1.5–2 h. AA and PG stand for amino acid and protecting group, respectively. All AAs in **1a**–**c**, **2a**–**c**, and **4a**–**c** were protected.

We have reported water-soluble complexes of C_60_ with a nontoxic and nonionic polymer, poly(vinylpyrrolidone) (PVP) [25] and applied these to several in vitro biological assays to report DNA photocleavage [26] and related ROS generation [27–28], antimicrobial photoactivity [29], chondrogenesis-promoting activity [30–31], photocytotoxicity [32–33], and GST enzyme inhibition [34]. For the covalent functionalization, we have previously developed a versatile and convenient biscarboxylic acid-substituted C_60_ derivative (**3**, Figure 1) [35], which was prepared via the Prato reaction [36]. We used this derivative **3** as a starting material and synthesized a series of water-soluble C_60_ and C_70_ derivatives by covalently attaching biocompatible water-soluble polymers, such as polyethylene glycol (PEG) [37–38] and PVP [39]. Although these C_60_– and C_70_–polymer conjugates revealed high water-solubility, it was found that they, especially the PEG conjugates, formed micelle-like aggregations in aqueous solution, as observed by dynamic light scattering (DLS), cryoTEM, and concentration-dependent surface tension measurements [40]. Despite the small size (≈10 nm in hydrodynamic diameter), the aggregation of these fullerene moieties was not ideal for biological applications as photodynamic therapy photosensitizers (PDT PSs) [41] and magnetic resonance imaging contrast agents (MRI CAs) [42], which are the most relevant topics in fullerene biological studies. Aggregated fullerenes in the micelle structure may cause self-quenching of the excited state of PS fullerenes or may inhibit the approach of bulk water molecules to the fullerene core and hamper water exchange – an important factor for enhancement in MRI. Well-dispersed water-soluble C_60_ derivatives exhibiting less aggregation are in high demand.

To address the challenge mentioned above, we developed highly water-soluble C_60_–peptide conjugates in this study. In addition to the water solubility introduced by the peptides, these conjugates have a superior biocompatibility compared to those with synthetic polymers, such as PEG and PVP. We utilized the previously reported biscarboxylic acid derivative **3**, which was suitable for the coupling to peptides on resin, prepared by solid-phase peptide synthesis (SPPS) [35]. The detailed conditions for the amide-forming reaction were optimized using biscarboxylic acid-substituted C_60_ derivative **3** and a similar peptide with a primary amine derived from γ-aminobutyric acid (GABA) at the N*-*terminus of the Lys pentamer peptide on resin. Using the optimal reaction conditions, three types of hydrophilic peptide pentamers on resin, oligo-Lys (**2a**), oligo-Glu (**2b**), and oligo-Arg (**2c**), were subjected to the reaction with **3** for the synthesis of C_60_–peptide conjugates **5a**–**c** (Figure 1).

## Results and Discussion

### Syntheses of C_60_–oligopeptides **5a–c**

The oligopeptides **2a**–**c** were synthesized on resin using Fmoc-protected amino acids with a standard SPPS method (Figure 1a) [43]. A moderate loading (0.4 mmol⋅g^−1^) of the initial amino acid was used. After synthesizing the pentamer of Lys on resin, a GABA residue was attached to the N-terminus of the peptide in order to provide a less-hindered primary amine, enabling an efficient amide conjugation reaction with biscarboxylic acid-substituted C_60_ derivative **3**.

Compound **3** was prepared by Prato reaction of C_60_ and an *N*-glycine derivative and subsequent deprotection of the *t-*Bu group under acidic conditions, without affecting the C_60_ cage (Figure 1b). Challenges in this step included finding suitable conditions to conjugate one C_60_ moiety and two peptide anchors on the resin. Preliminarily, the reaction conditions were optimized using a similar peptide (GABA-(Lys)_5_-peptide-PEG) on resin for the reaction with compound **3**. Initial trials, using 2-(1*H*-7-azabenzotriazol-1-yl)-1,1,3,3-tetramethyluronium hexafluorophosphate (HATU) and *N*-methylmorpholine (NMM), respectively, as a coupling reagent and a base, and 5 equiv of peptide on resin (rink amide MBHA) relative to **3**, provided a rather low yield (13%, isolated by HPLC), which was increased to 24% by changing the solid phase to 2-chlorotrityl chloride resin. By replacing the base with *N*,*N*-diisopropylethylamine (DIPEA), the yield was slightly increased to 28%, which became higher (32%) when HBTU was used as a coupling reagent. Finally, use of a greater excess (6.0 equiv) of peptide on resin relative to **3**, and a combination of HBTU and DIPEA as the coupling reagent and base, provided the C_60_–peptide conjugate in an isolated yield of 41% (based on the used compound **3**). The yield of C_60_–peptide conjugate formation decreased when using fewer equivalents of peptide on resin relative to compound **3**, providing as a byproduct the C_60_–peptide conjugate connected to only one peptide anchor.

Based on the optimized reaction conditions outlined above, the conjugation of biscarboxylic acid-substituted C_60_ derivative **3** and the peptides on resin **2a**–**c** were performed by SPPS to provide the C_60_–oligopeptides on resin **4a**–**c** (Figure 1a). Subsequently, the last step of the reaction – the cleavage of the C_60_–peptide conjugate from the resin and the simultaneous deprotection of the amino acid residues – provided C_60_–peptide conjugates **5a**–**c** with fully deprotected peptides.

The syntheses of C_60_–oligo-Lys (**5a**), C_60_–oligo-Glu (**5b**), and C_60_–oligo-Arg (**5c**) were confirmed by HRMS (Figures S2, S11, and S18, Supporting Information File 1). While C_60_–oligo-Lys (**5a**) was successfully observed by HRESIMS in a charged state of 3+ (Figure S2, Supporting Information File 1), C_60_–oligo-Glu (**5b**) was confirmed by HRMS–MALDI (Figure S11, Supporting Information File 1) due to insolubility in the acidic eluent generally used for HRESIMS (a mixture of MeOH and water containing 0.1% formic acid). C_60_–oligo-Arg (**5c**), which was not sufficiently soluble in most of the solvents, was slightly soluble in the acidic eluent, so that HRESIMS analysis provide HRMS data for a charged state of 4+ (Figure S18, Supporting Information File 1).

C_60_–oligo-Lys (**5a**), with sufficient solubility in polar solvents, was isolated by reversed-phase HPLC. C_60_–oligo-Glu (**5b**), which was soluble only in basic aqueous solution, could not be isolated by HPLC, especially in the presence of an oligo-Glu impurity, and was purified only after spin filtration. C_60_–oligo-Arg (**5c**) was not soluble in any solvent and could not be further purified.

### Solubility in water

**Figure 2:**
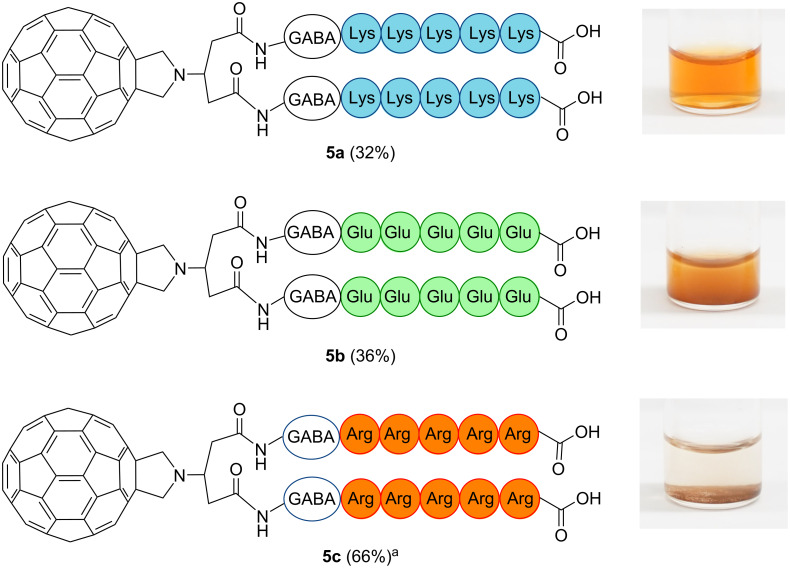
Structure of C_60_–oligo-Lys (**5a**), C_60_–oligo-Glu (**5b**), and C_60_–oligo-Arg (**5c**) and images of dissolved or dispensed compounds in Milli-Q^®^ water (pH 7.0). ^a^Crude yield.

The water solubility of C_60_–peptide conjugates **5a–c** was tested after the removal of any remaining solvent traces by lyophilization. Upon addition of Milli-Q^®^ water (pH 7.0), C_60_–oligo-Lys (**5a**) was immediately and thoroughly solubilized. In contrast, the other conjugates, C_60_–oligo-Glu (**5b**, purified) and C_60_–oligo-Arg (**5c**, crude), did not produce transparent solutions in water at neutral pH value even by sonication (Figure 2). While C_60_–oligo-Glu (**5b**) was soluble in buffer with a higher pH value (>8.3), C_60_–oligo-Arg (**5c**) was not soluble in most polar and nonpolar solvents. In addition, **5a** was not highly soluble in most nonpolar solvents, including toluene and CH_2_Cl_2_, but slightly soluble in other polar solvents, including MeOH and DMSO.

**Figure 3:**
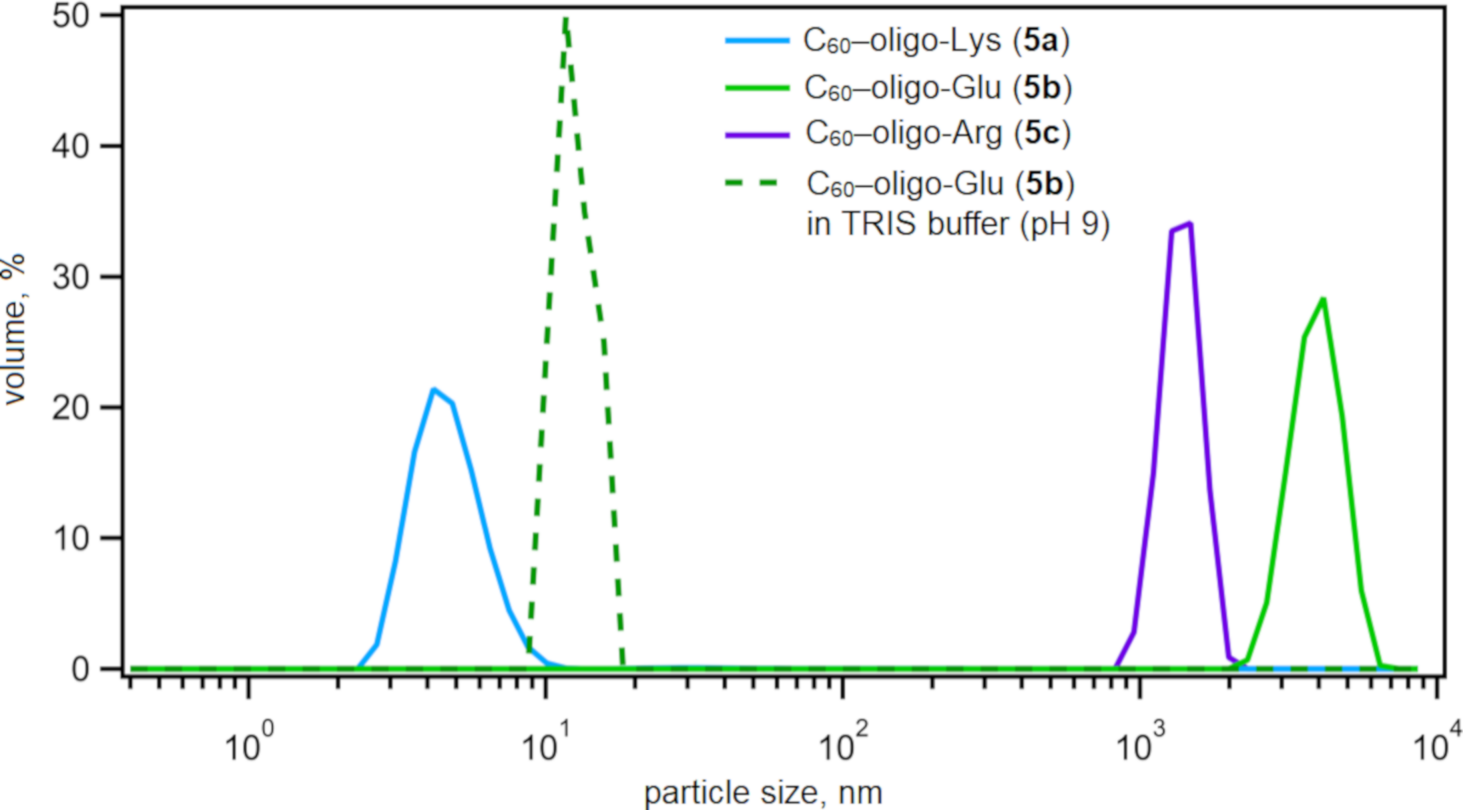
DLS diagrams of C_60_–peptide conjugates **5a** (1 mM, in Milli-Q^®^ water), **5b** (1 mM, in Milli-Q^®^ water or in pH 9.0 TRIS buffer), and **5c** (1 mM, in Milli-Q^®^ water). Particle size: mean, nm (polydispersity index, PDI): **5a**: 4.8 (0.757), **5b**: 3974 (0.906), **5c** (crude): 1382 (0.115) in Milli-Q^®^ water and **5b**: 11.8 (1.000) in pH 9.0 TRIS buffer. Milli-Q^®^ water was pH 7.0.

The solubility of C_60_–peptide conjugates **5a–c** was in line with DLS data of the aqueous solutions or dispersions. While **5a** (blue line) revealed an extremely small hydrodynamic diameter (<10 nm) by DLS, **5b** (green line) and **5c** (purple line) revealed the presence of larger aggregates in water (pH 7.0), with a hydrodynamic diameters larger than 1 µm (Figure 3). In buffer at a higher pH value (e.g., pH 9, TRIS buffer), C_60_–oligo-Glu (**5b**) showed much smaller aggregation (dotted green line, ≈12 nm), providing a transparent solution, while C_60_–oligo-Arg (**5c**) remained insoluble over the tested pH value range (4.0–9.2). This was presumably due to the strong cation–π interactions between the cationic Arg moieties and the aromatic C_60_, which is generally enhanced in polar environments [44–46]. The list of the solvents used to solubilize the molecules is summarized in Table S1, Supporting Information File 1.

### Spectral characterizations of **5a** and **5b**

**Figure 4:**
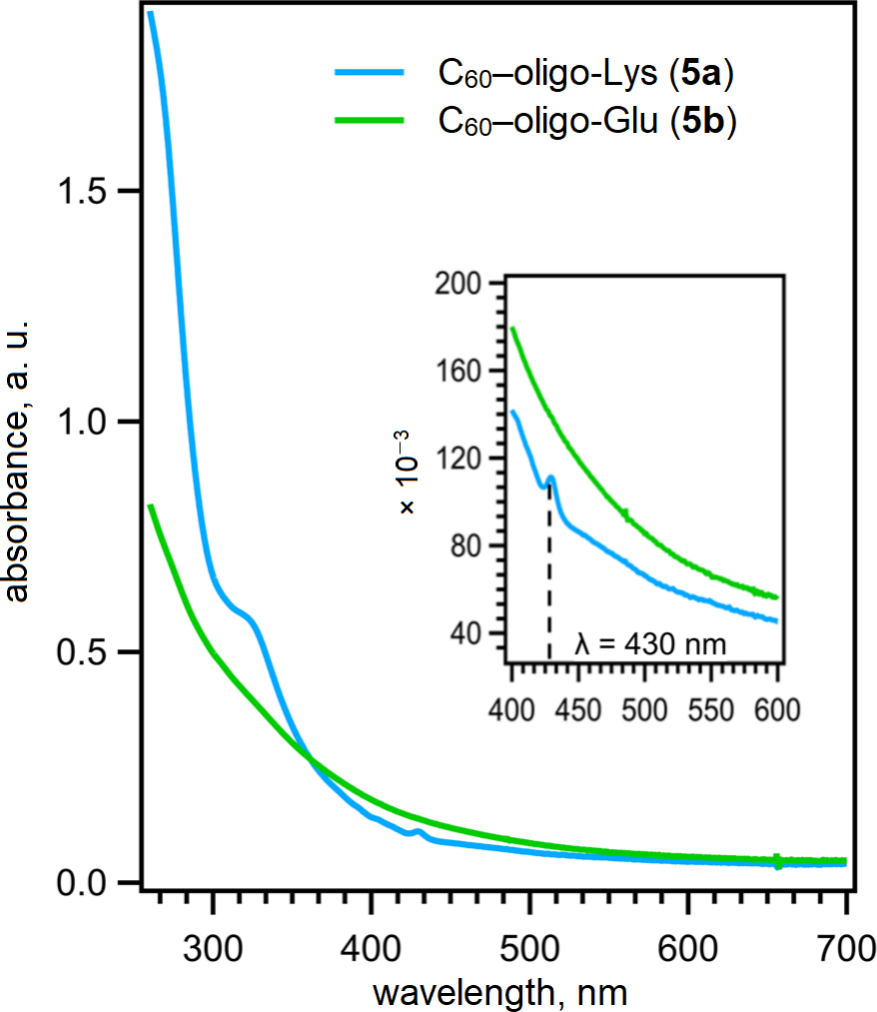
UV–vis spectra of C_60_–peptide conjugates **5a** and **5b** (20 μM in Milli-Q^®^ water for **5a** and in pH 9.0 TRIS buffer for **5b**).

The absorption spectra of C_60_–oligo-Lys (**5a**) and C_60_–oligo-Glu (**5b**) were recorded in Milli-Q^®^ water (pH 7.0) and in TRIS buffer (pH 9.0), respectively (Figure 4). The spectrum of C_60_–oligo-Lys (**5a**) was in good agreement with that typically observed for C_60_ monoadduct derivatives [47]. The electronic spectra of the fulleropyrrolidines were characterized by notable π–π* absorption in the UV region. Additionally, **5a** exhibited broad absorption in the visible region with relatively low intensity as well as a distinctive sharp peak at around 430 nm [48]. However, those features were not observed in the spectrum of C_60_–oligo-Glu (**5b**), presumably due to the aggregation [32].

**Figure 5:**
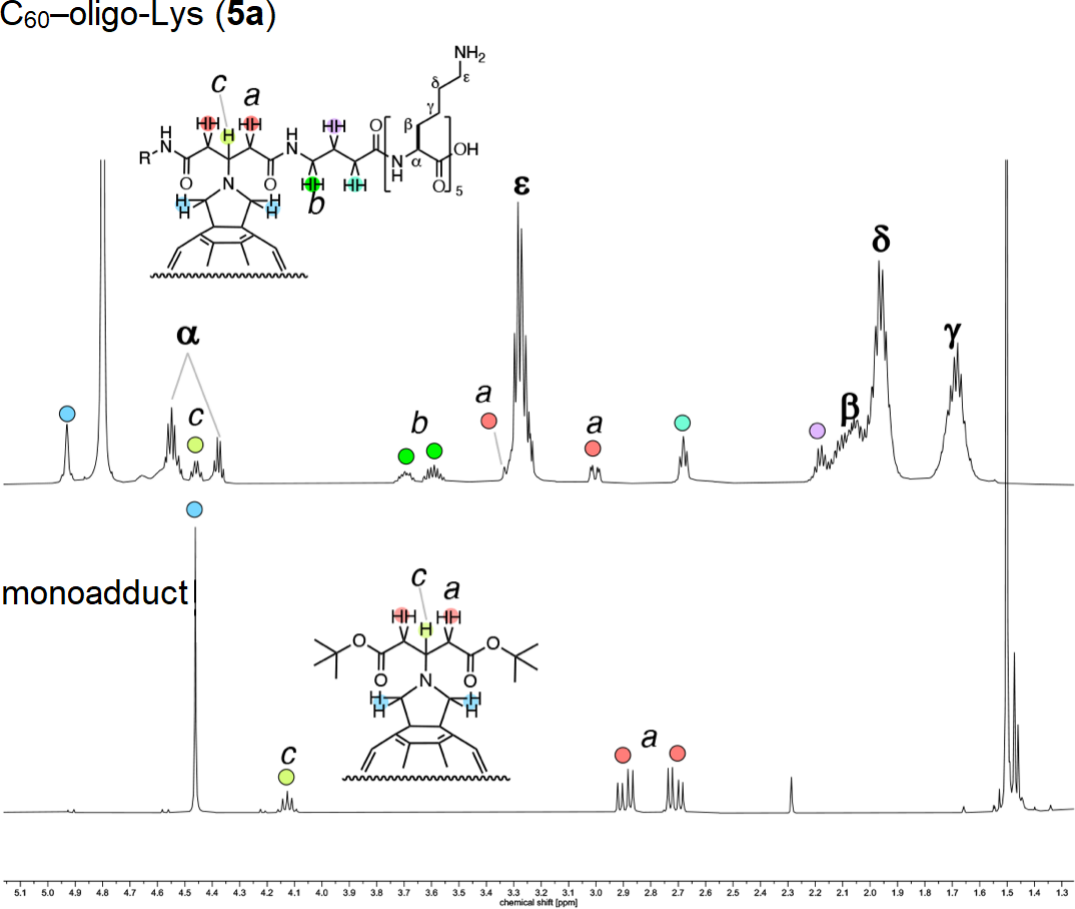
^1^H NMR spectrum of C_60_–peptide conjugate **5a** in D_2_O (above) and of the precursor monoadduct in CDCl_3_ (bottom) at 600 MHz.

The ^1^H NMR spectrum of C_60_–oligo-Lys (**5a**) was recorded in D_2_O. As shown in Figure 5, the spectrum of **5a** (upper spectrum) clearly shows peaks for protons corresponding to the fulleropyrrolidine part, which are in line with the peaks in the spectrum of the C60 Prato monoadduct (lower spectrum), linker part, and oligo-Lys side chain part (α, β, γ, δ, and ε). The observed splitting of the protons a and b was presumably due to a diastereotopic effect of the methine proton c, similar to the spectrum of the monoadduct.

**Figure 6:**
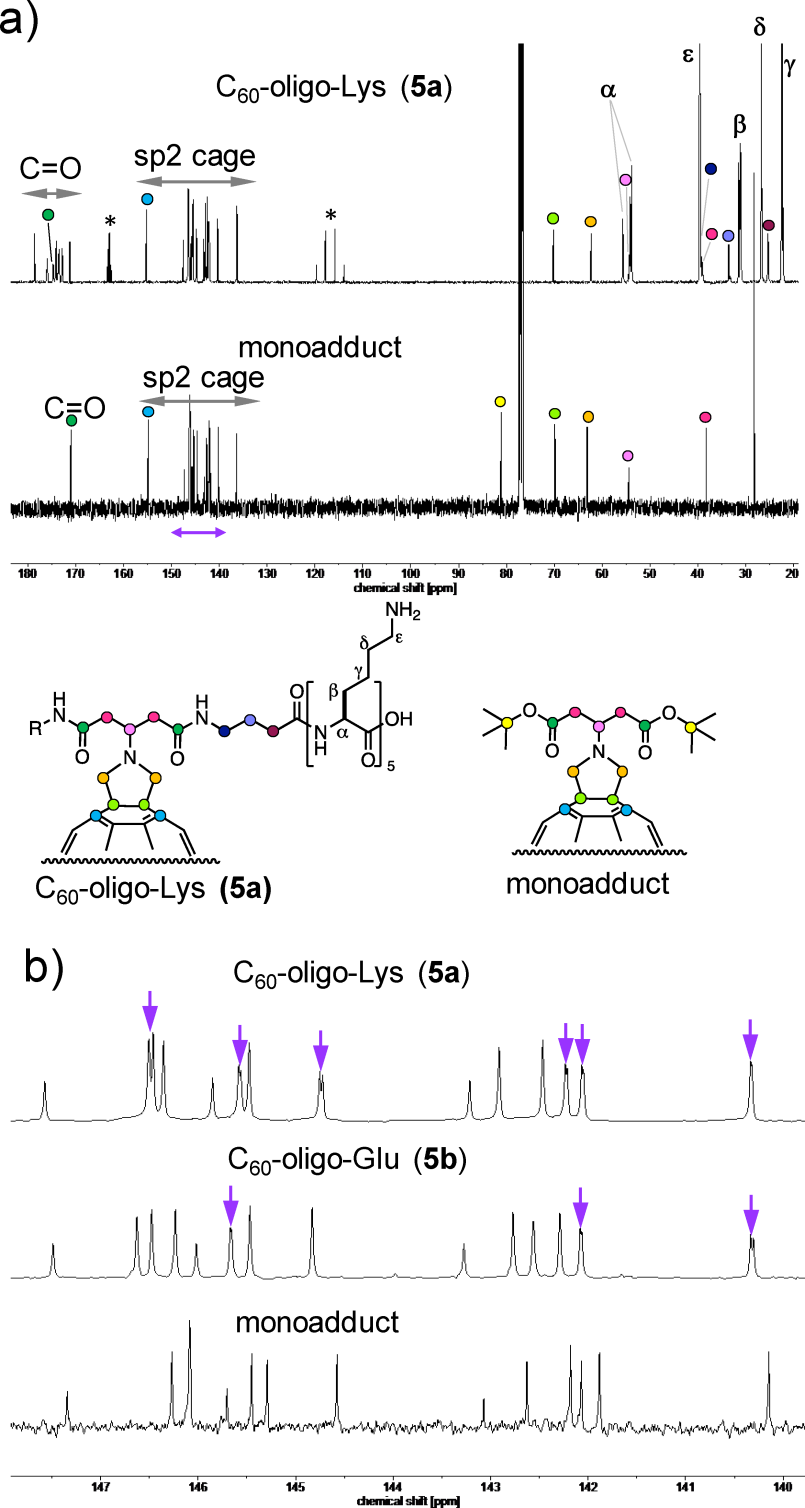
^13^C NMR spectrum of C_60_–peptide conjugate **5a** in D_2_O and of the precursor monoadduct in CDCl_3_ at 150 MHz (a) and expansion of the sp^2^ carbon region (b). The asterisks in (a) correspond to a TFA impurity. Purple arrows in (b) indicate the split peaks. The ^1^H NMR spectrum of purified **5b** was measured in D_2_O with 2% NaOD (Figure S12, Supporting Information File 1). ^13^C NMR analysis of the same sample was not possible due to the high ionic strength of the solution. NMR characterization was performed using a crude sample of **5b**, with the penta-Glu impurity being soluble in pyridine-*d*_5_ (Figures S13–S17, Supporting Information File 1).

Figure 6a shows the ^13^C NMR spectra of **5a** in D_2_O and of the monoadduct in CDCl_3_. Together with the ^1^H NMR, COSY, HSQC, and HMBC spectra (Figures S3–S9, Supporting Information File 1), all peaks corresponding to the pyrrolidine part, linker part, and oligo-Lys part were assigned as shown in the chemical structure. In the sp^2^ region of **5a** (Figure 6a, top), 17 signals (1C × 3 + 2C × 13) were observed similarly to the monoadduct (Figure 6a, bottom), which corresponds to the fullerene core in a characteristic manner for a *C*_2_*_v_*-symmetric structure with a [6,6]-addition pattern. In the expanded spectrum of **5a** in the aromatic region (Figure 6b, top), several intense signals (corresponding to 2C) were observed as split peaks (highlighted by purple arrows). A similar situation was observed in the expanded spectrum of **5b** (Figure 6b, middle, measured in pyridine). This phenomenon suggests a symmetry break in the carbon cage moiety of the C_60_–peptide conjugate upon the addition of chiral peptide anchors to the C_60_ core. Together with the HRESIMS results (Figure S2, Supporting Information File 1), it was confirmed that the highly water-soluble compound **5a** was successfully synthesized.

### ^1^O_2_ generation under visible light irradiation

**Figure 7:**
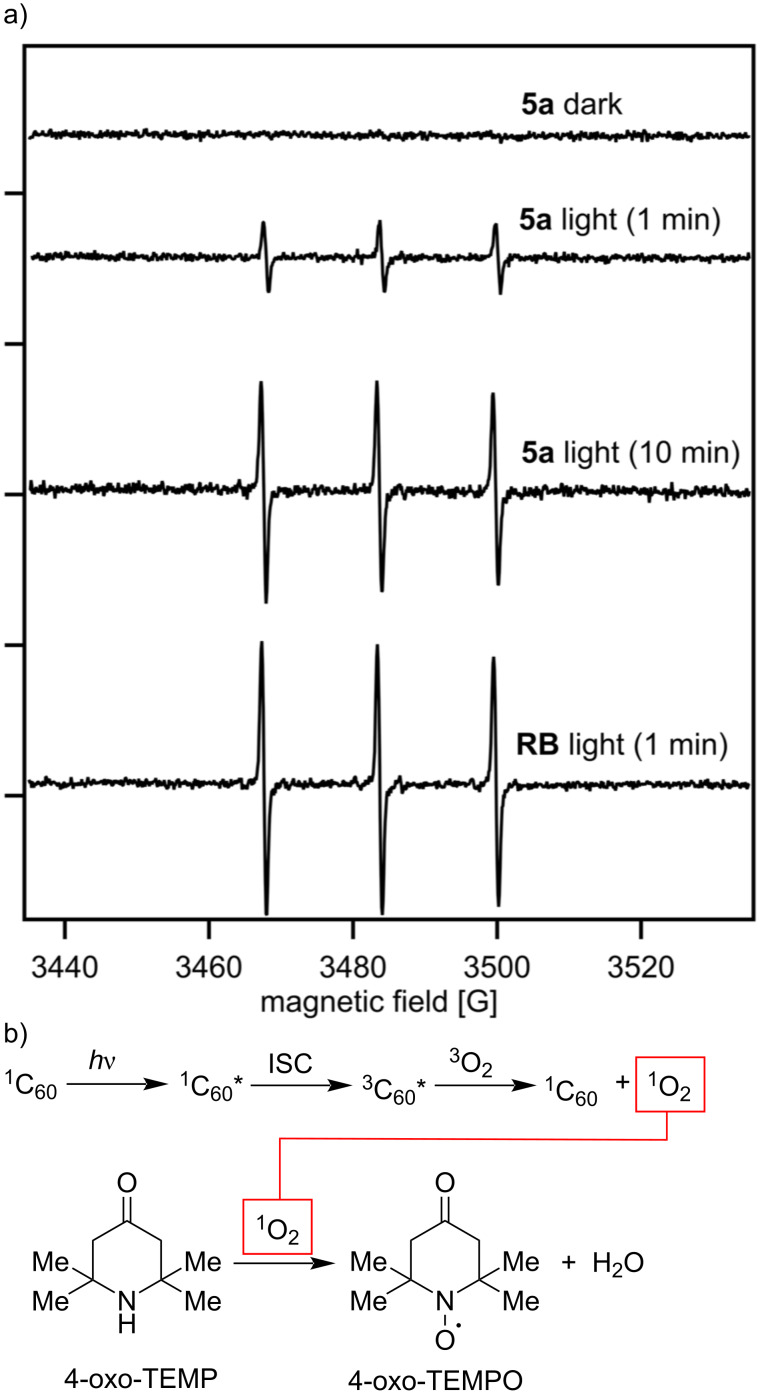
a) X-band ESR spectra of the 4-oxo-TEMP adduct with ^1^O_2_ generated by C_60_–oligo-Lys (**5a**) and rose bengal (RB), respectively, in aqueous solutions under irradiation with a green LED lamp (527 nm) for 1 min or 10 min. PS: 40 μM, 4-oxo-TEMP: 80 mM, in 60 mM phosphate buffer (pH 7.0). Measurement conditions: temperature 296 K, microwave frequency 10.03 GHz, microwave power 10 mW, receiver gain 5.0 × 10^4^, modulation amplitude 1.00 G, modulation frequency 100 KHz, sweep time 83.89 s, number of scans: 1. b) Scheme for the photoinduced ^1^O_2_ generation by C_60_ and reaction with spin trapping reagent 4-oxo-TEMP to form 4-oxo-TEMPO.

To preliminarily evaluate the synthesized C_60_–oligo-Lys (**5a**) as a PS, generation of singlet oxygen was measured by the ESR spin trapping method under irradiation of visible light (527 nm green LED). 4-Oxo-TEMP was used as a spin trapping reagent to form an adduct with ^1^O_2_, i.e., 4-oxo-TEMPO, which was observed by ESR (Figure 7b). As shown in Figure 7a, upon visible light irradiation, three peaks corresponding to 4-oxo-TEMPO were observed in the solution of C_60_–oligo-Lys (**5a**), similar to the results with rose bengal, a standard compound for ^1^O_2_ generation. By taking into account that the absorption intensity of **5a** at 527 nm used for the photoirradiation was ≈10 times smaller than that of rose bengal, it was suggested that ^1^O_2_ was sufficiently present in the solution of **5a**.

## Conclusion

Starting from biscarboxylic acid-substituted fulleropyrrolidine derivative **3**, the three C_60_–oligopeptides **5a**–**c** were synthesized through SPPS. Between these conjugates, C_60_–oligo-Lys (**5a**) had sufficient solubility and a very small hydrodynamic diameter in a neutral aqueous medium, as shown by DLS analysis. Visible-light-induced ^1^O_2_ generation by C_60_–oligo-Lys (**5a**) was confirmed by ESR spin trapping. This suggests the high potential of **5a** as a basis for a fullerene-derived PS in biological applications.

## Experimental

### Synthesis of oligopeptides on resin **1a–c**

The oligopeptides on resin **1a–c** were synthesized via a general SPPS protocol. The first addition of Fmoc-AA(PG)-OH to the 2-chlorotrityl resin was performed in the presence of DIPEA (2 equiv) in CH_2_Cl_2_, followed by capping with a CH_2_Cl_2_/MeOH/DIPEA (18:2:1, v/v) mixture to quench any remaining unreacted chlorotrityl moieties on the resin surface. Subsequently, four additional Fmoc-AA(PG)-OH residues and one Fmoc-GABA-OH residue were added to the resin to provide the peptides on resin **1a–c**. Each coupling step was carried out in the presence of HCTU (4 equiv) and NMM (8 equiv) in DMF. Each Fmoc deprotection step of the peptide N-terminus was conducted by the repeated treatment of the peptide on resin with 20% piperidine in DMF (2 × 10 min). After each coupling reaction, the resin was washed with DMF.

### Synthesis of C_60_–peptide conjugates **5a–c**

The synthetic details and corresponding spectra for the C_60_–peptide conjugates **5a–c** are shown in Supporting Information File 1. The optimization of the conditions for the reaction between the peptides on resin and fulleropyrrolidine **3** are described in the Results and Discussion section above. These were used to prepare the C_60_–peptide conjugates on resin **4a–c** from **2a–c**. The C_60_–oligopeptides **5a–c** were obtained by cleavage from resin and simultaneous deprotection of the PGs on the amino acid side chains through the addition of by the addition of a mixture of TFA and TIPS in water. The most soluble conjugate, C_60_–oligo-Lys (**5a**), was purified by reversed-phase HPLC, while C_60_–oligo-Glu (**5b**) was purified by spin filtration.

C_60_–oligo-Lys (**5a**) was obtained in a yield of 32% for the total peptide synthesis and characterized by HRESIMS. HRESIMS (*m*/*z*): [M + 3H]^3+^ calcd for C_135_H_148_N_23_O_16_, 782.3819; found, 782.3821.

C_60_–oligo-Glu (**5b**) was obtained in a yield of 36% for the total peptide synthesis and characterized by HRMS–MALDI. HRMS–MALDI (*m*/*z*): [M + H]^+^ calcd for C_125_H_96_N_13_O_36_, 2354.6075; found, 2354.6008.

C_60_–oligo-Arg (**5c**) was obtained in a crude yield of 66% for the total peptide synthesis and characterized by HRESIMS. HRESIMS (*m*/*z*): [M + 4H]^4+^ calcd for C_135_H_149_N_43_O_16_, 657.0536; found, 657.0540.

## Supporting Information

File 1 Details for the synthesis of **5a–c** and intermediates as well as spectral data.

## Data Availability

All data that supports the findings of this study is available in the published article and/or the supporting information to this article.
