# Supplementary material for: Synthesis and characterization of water-soluble C60–peptide conjugates
Source: Beilstein J Org Chem. 2024 Apr 12;20:777–86. doi: 10.3762/bjoc.20.71 (PMC11022410; doi:10.3762/bjoc.20.71)
Supplement: File 1 — Details for the synthesis of 5a–c and intermediates as well as spectral data. [file Beilstein_J_Org_Chem-20-777-s001.pdf]

BEILSTEIN JOURNAL OF ORGANIC CHEMISTRY

Supporting Information

for

Synthesis and characterization of water-soluble C_{60}–peptide conjugates

Yue Ma, Lorenzo Persi and Yoko Yamakoshi

Beilstein J. Org. Chem. **2024,** *20,* 777–786. doi:10.3762/bjoc.20.71

Details for the synthesis of 5a–c and intermediates as well as spectral data

License and Terms: This is a supporting information file under the terms of the Creative Commons Attribution License (https://creativecommons.org/licenses/by/4.0). Please note that the reuse, redistribution and reproduction in particular requires that the author(s) and source are credited and that individual graphics may be subject to special legal provisions.
The license is subject to the *Beilstein Journal of Organic Chemistry* terms and conditions: (https://www.beilstein-journals.org/bjoc/terms)

Table of contents

Synthesis of C_{60}–oligopeptides **5a**–**c** S1

DLS S20

Solubility of C_{60}–oligopeptides **5a**–**c** S20

Detection of photoinduced $^1O_2$ generation by ESR spin trapping S21

Synthesis of C_{60}-oligopeptides 5a–c

General

HRMS was performed on either a Bruker Daltonics maXis ESI–QTOF spectrometer or on a Bruker Daltonics solariX spectrometer. HPLC was performed using a JASCO PU-2080 Plus HPLC pump, a JASCO MD2018 Plus detector, and a ChromNAV Chromatography Data System. UV–vis spectra were recorded on a Varian Cary-500 spectrophotometer. Solvents were purchased from Acros Organics. Water (Milli-Q® water) was obtained from a Millipore purification system. C_{60} was purchased from SES Research Inc. Fmoc-protected amino acids were purchased from Novabiochem. Trityl resin was purchased from Iris Biotech GmbH. Trifluoroacetic acid (TFA), 2-(1*H*-benzotriazole-1-yl)-1,1,3,3-tetramethyluronium hexafluorophosphate (HBTU), *O*-(1*H*-6-chlorobenzotriazole-1-yl)-1,1,3,3-tetramethyluronium hexafluorophosphate (HCTU), *N*-methylmorpholine (NMM), piperidine, triisopropylsilane (TIPS), and diisopropylethylamine (DIPEA) were purchased from Sigma-Aldrich.

Scheme S1. Synthesis of C_{60}–oligo-Lys (**5a**).

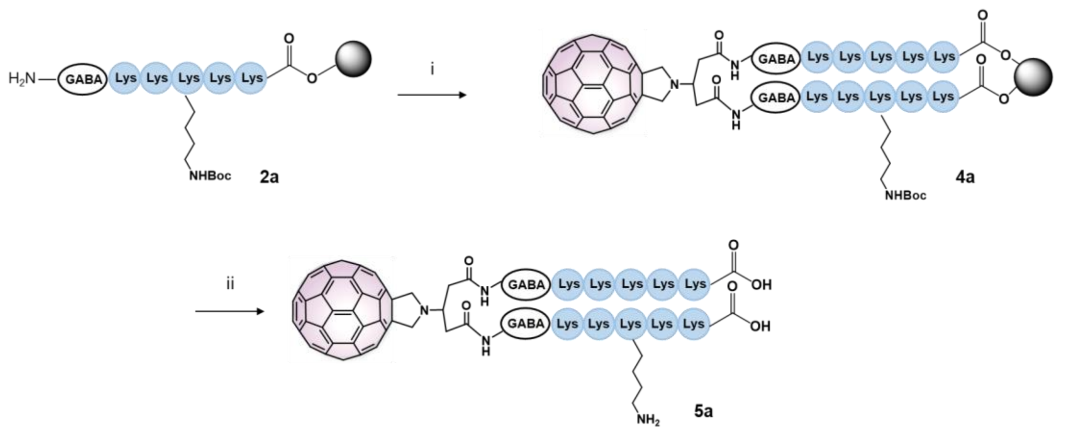

Reagents and conditions: i) **3**, HBTU, DIPEA, in DMF, rt, overnight, ii) TFA/TIPS/H_2O, rt, 1.5–2 h.

Peptide on resin 2a

The peptide was prepared on chlorotrityl resin (loading of 0.293 mmol·g^{-1}, 500 mg resin). The resin was subjected to the first addition of Fmoc-Lys(Boc)-OH. The automated peptide elongation was carried out on a Biotage Syro I peptide synthesizer according to the general SPPS methods. The subsequent reactions were carried out with the following Fmoc-protected amino acids (4 equiv): Fmoc-Lys(Boc)-OH, Fmoc-Lys(Boc)-OH, Fmoc-Lys(Boc)-OH, and Fmoc-GABA-OH. Each coupling on the resin was carried out in the presence of HCTU (4 equiv) and NMM (8 equiv) in DMF. Fmoc deprotection of each step was conducted by the repeated treatment of Fmoc-protected peptide on resin with 20% piperidine in DMF (two times for 10 min each). After each coupling reaction, the resin was washed with DMF. Fmoc-GABA-OH was coupled manually in the presence of 4 equiv of Fmoc-GABA-OH, 4 equiv of HCTU, and 8 equiv of NMM and deprotected by 20% piperidine in DMF to provide peptide on resin **2a**.

C_{60}–peptide on resin 4a

To the peptide on resin **2a** with a deprotected terminal amine (NH_2-GABA-KKKKK(Boc)-resin), fullerene monoadduct **3** (13.3 mg, 0.015 mmol) in DMF (3.5 mL), HBTU (58.1 mg, 0.153 mmol), and DIPEA (37 μL,

0.335 mmol) were added. The mixture was agitated overnight at room temperature, filtered, and washed with DMF and CH_2Cl_2 5 times to provide C_{60}−peptide on resin **4a**.

C_{60}−oligo-Lys (5a)

C_{60}−poly-Lys with protective groups on resin **4a** was treated with a mixture of TFA/TIPS/H_2O (95:2.5:2.5, v/v) for 1.5−2 h, and the resin was removed by filtration. The filtrate was concentrated in vacuo, triturated with Et_2O, and centrifuged to obtain the crude peptide. The crude peptide was dissolved in a mixture of H_2O/CH_3CN (1:1, 0.2 mL). The peptide was purified by semipreparative HPLC (column: Shiseido Capcell Pak C18 (20 mm × 250 mm), eluent: an isocratic system of CH_3CN/H_2O 30:70 for 5 min, then a gradient system of CH_3CN/H_2O (30:70 to 70:30) over 30 min, all in the presence of 0.1% TFA, flow rate: 10 mL·min^{-1}, detection: 365 nm) to provide the light brown solid **5a** (11.2 mg, 4.7 µmol, yield = 32%).

^{1}H NMR (600 MHz, D_2O): δ 1.55–1.75 (m, 20H, Lys side chain $CH_2C\underline{H}_2CH_2CH_2NH_2$), 1.8–1.98 (m, 20H, Lys side chain $CH_2CH_2C\underline{H}_2CH_2NH_2$), 1.98-2.1 (m, 20H, Lys side chain $C\underline{H}_2CH_2CH_2CH_2NH_2$), 2.1-2.17 (m, 4H, GABA $NHCH_2C\underline{H}_2CH_2CO$), 2.65 (t, J = 7.8 Hz, 4H, GABA $NHCH_2CH_2C\underline{H}_2CO$), 2.98 (dd, J = 14.8, 4.7 Hz, 2H, Prato adduct $CH_2CH(NH)C\underline{H}_2$), 3.17 – 3.27 (m, 20H, Lys side chain $CH_2CH_2CH_2C\underline{H}_2NH_2$), 3.27-3.31 (m, 2H, Prato adduct $C\underline{H}_2CH(NH)CH_2$), 3.56 (m, 2H, GABA $NHC\underline{H}_2CH_2CH_2CO$), 3.66 (m, 2H GABA $NHC\underline{H}_2CH_2CH_2CO$), 4.34 (m, 5H, Lys $COC\underline{H}$(sidechain)NH), 4.43 (td, J= 8.2, 5.7 Hz, 1H, Prato adduct $CH_2C\underline{H}(NH)CH_2$), 4.45-4.55 (m, 5H, Lys $COC\underline{H}$(sidechain)NH), 4.87 (s, 4H (suppressed due to presaturation), Prato adduct $C\underline{H}_2NH\underline{C}H_2$), 8.7 (s, 2H, Lys C-terminal $COO\underline{H}$); ^{13}C NMR (150 MHz, D_2O): δ 22.23-22.59 (Lys side chain $CH_2\underline{C}H_2CH_2CH_2NH_2$), 25.33 (GABA $NHCH_2\underline{C}H_2CH_2CO$), 25.43 (GABA $NHCH_2\underline{C}H_2CH_2CO$), 26.70-26.78 (Lys side chain $CH_2CH_2\underline{C}H_2CH_2NH_2$), 31.02-31.22 (Lys side chain $\underline{C}H_2CH_2CH_2CH_2NH_2$), 33.51 (GABA $NHCH_2CH_2\underline{C}H_2CO$), 33.52 (GABA $NHCH_2CH_2\underline{C}H_2CO$), 39.03 (GABA $NH\underline{C}H_2CH_2CH_2CO$), 39.52(Prato adduct $\underline{C}H_2CH(NH)\underline{C}H_2$), 39.61-39.56 (Lys side chain $CH_2CH_2CH_2CH_2\underline{N}H_2$), 53.81-55.73 (Lys $CO\underline{C}H$(sidechain)NH), 54.43 (Prato adduct $CH_2\underline{C}H(NH)CH_2$), 62.35 (Prato adduct $\underline{C}H_2NH\underline{C}H_2$), 70.21 ($sp^3$ $\underline{C}CH_2N$), 136-156 (sp2 cage region: 136.29 (4C), 140.32 (4C), 142.06 (4C), 142.22 (4C), 142.47 (4C), 142.91

(4C), 143.21 (2C), 144.74 (4C), 145.48 (4C), 145.57 (4C), 145.85 (2C), 146.36 (4C), 146.46 (4C), 146.51 (4C), 147.57 (2C), 155.27 (4C)), 171.12-178.45, (CONH); HRMS (ESI $^{+}$) *m/z* calcd for $[C_{135}H_{148}N_{23}O_{16}]^{3+}$: 782.3819, found: 782.3821 ($[M+3H]^{3+}$).

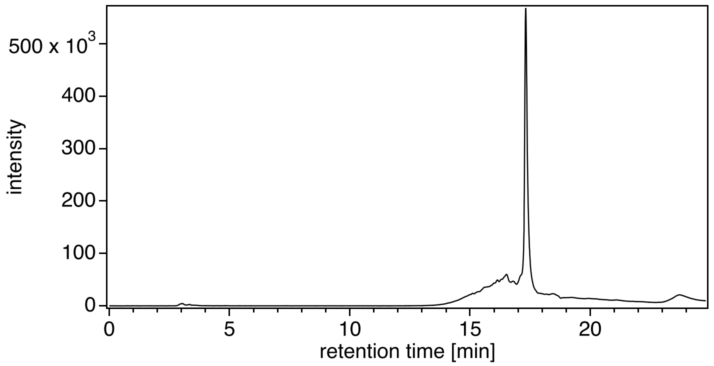

Figure S1. Analytical HPLC diagram of purified **5a** (column: Shiseido Capcell Pak C18 (20 mm × 250 mm), eluent: an isocratic system of CH_3CN/H_2O (5:95) for 3 min, a gradient system of CH_3CN/H_2O (5:95 to 95:5) over 14 min, an isocratic system of CH_3CN/H_2O (95:5) for 5 min, then an isocratic system of CH_3CN/H_2O (5:95), all in the presence of 0.1% TFA, flow rate: 10 mL·min^{-1}, detection: 365 nm).

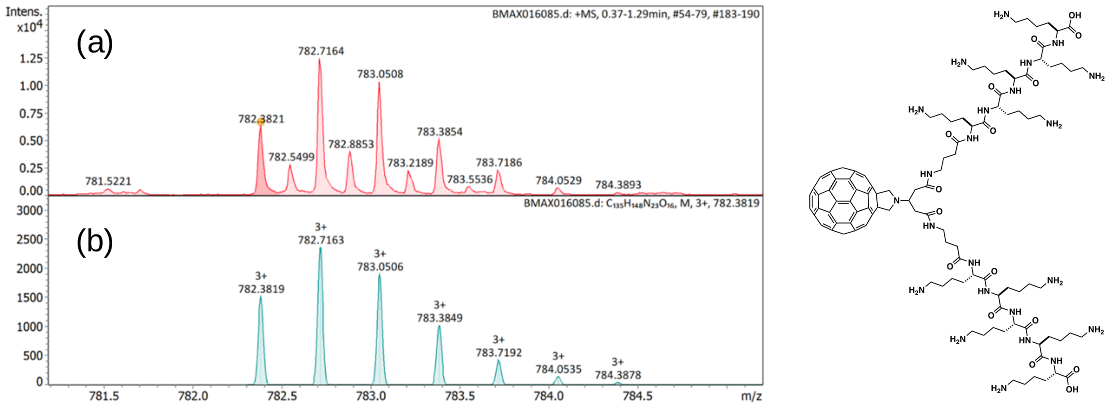

Figure S2. HRMS–ESI spectrum of **5a**, measured (a) and simulated (b), and chemical structure.

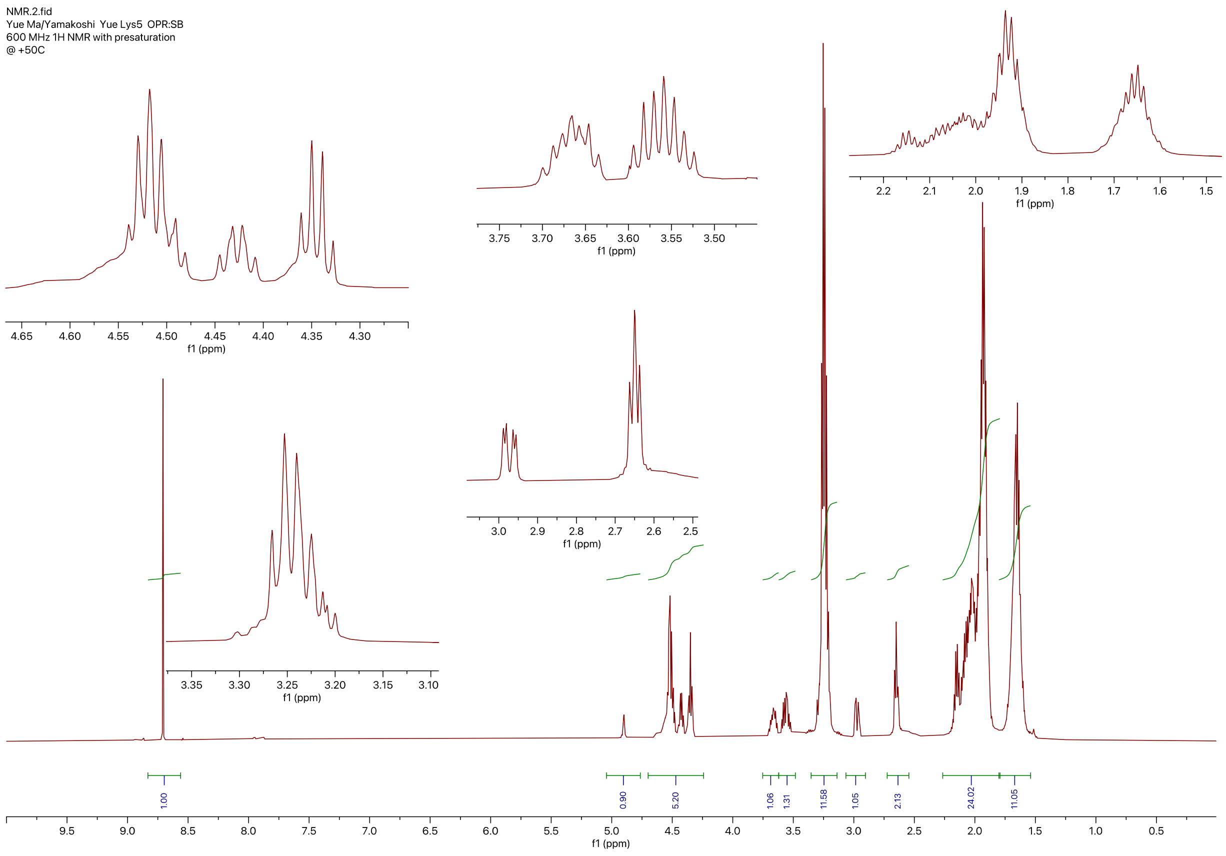

Figure S3. ^{1}H NMR spectrum of **5a** in D_2O (600 MHz).

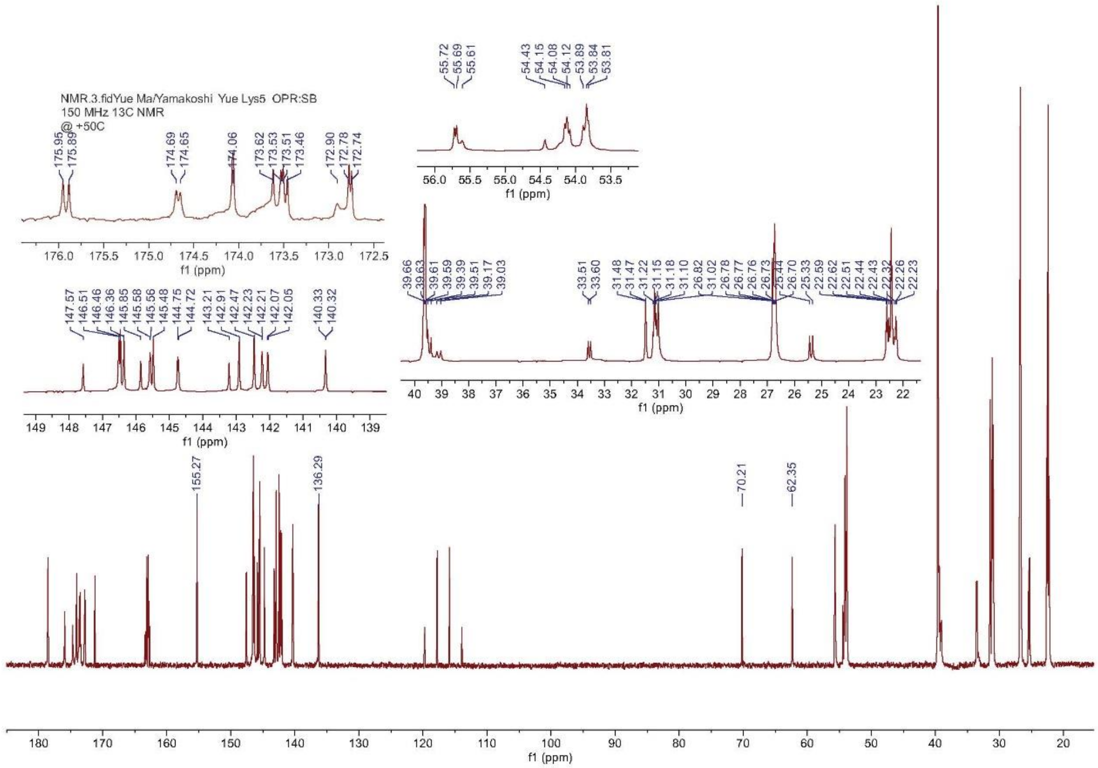

Figure S4. ^{13}C NMR spectrum of **5a** in D_2O (150 MHz).

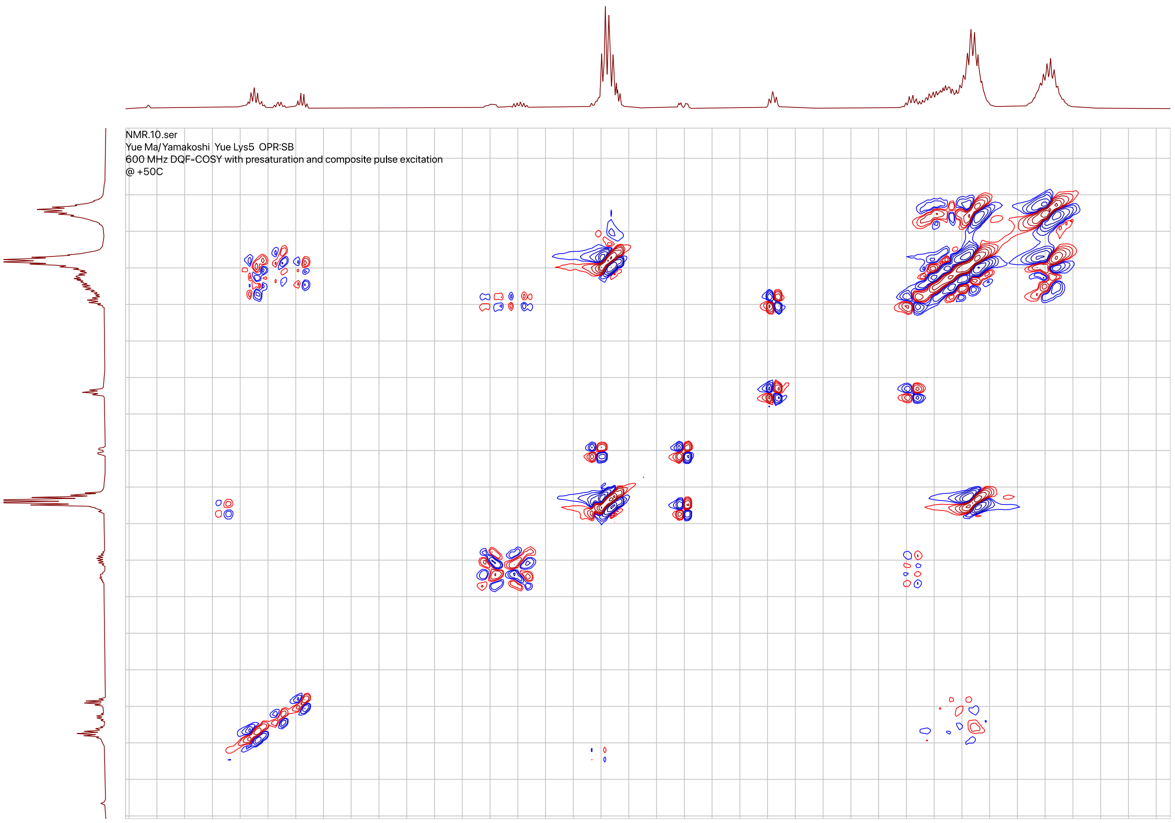

Figure S5. $^{1}H,^{1}H$-COSY spectrum of **5a** in D_2O.

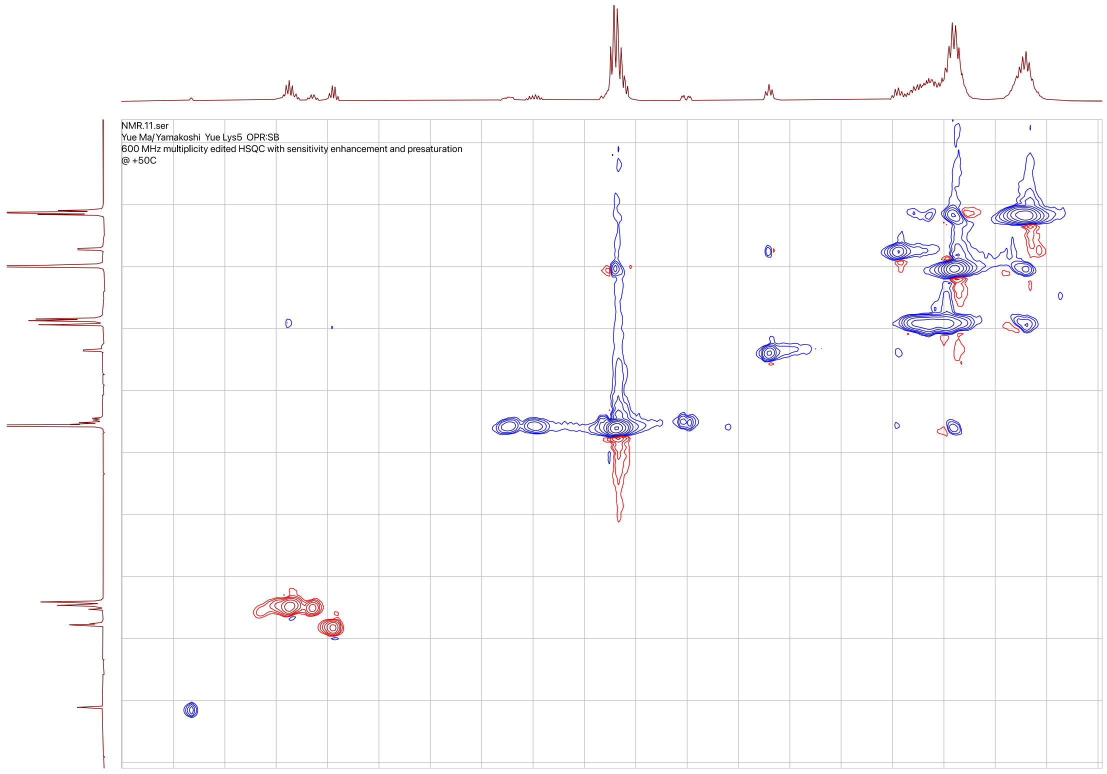

Figure S6. HSQC spectrum of **5a** in D_2O.

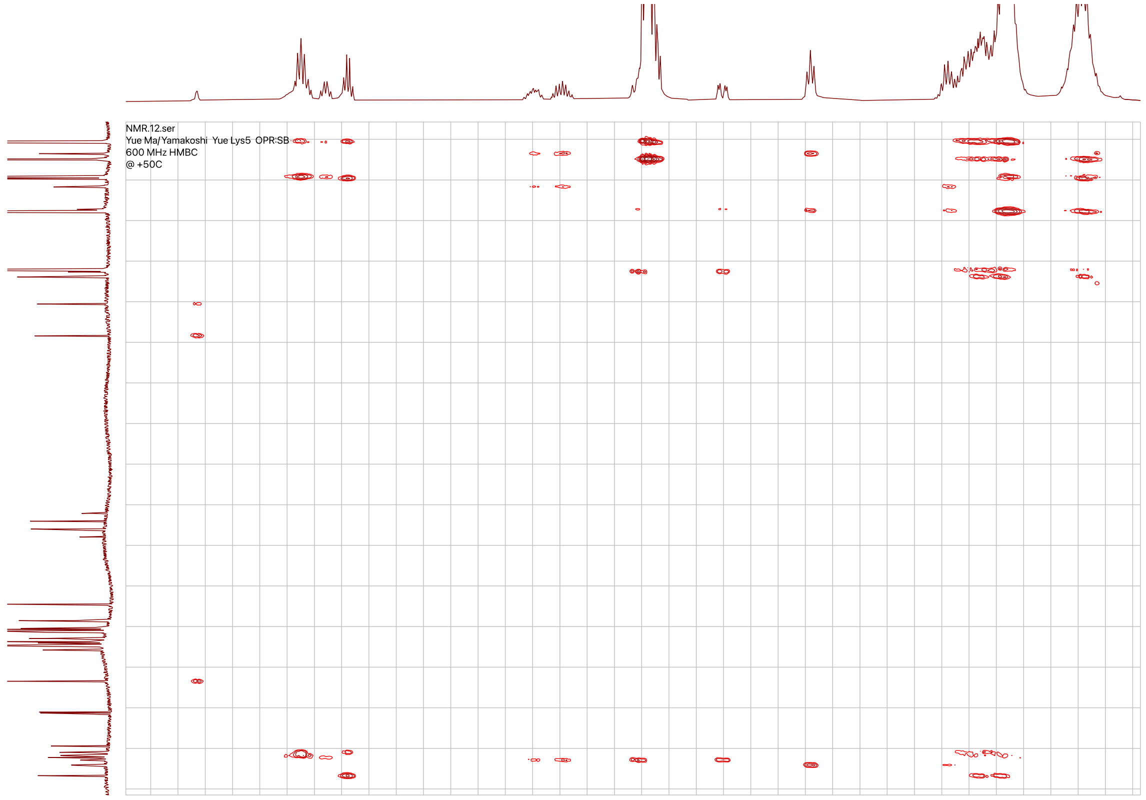

Figure S7. HMBC spectrum of **5a** in D_2O.

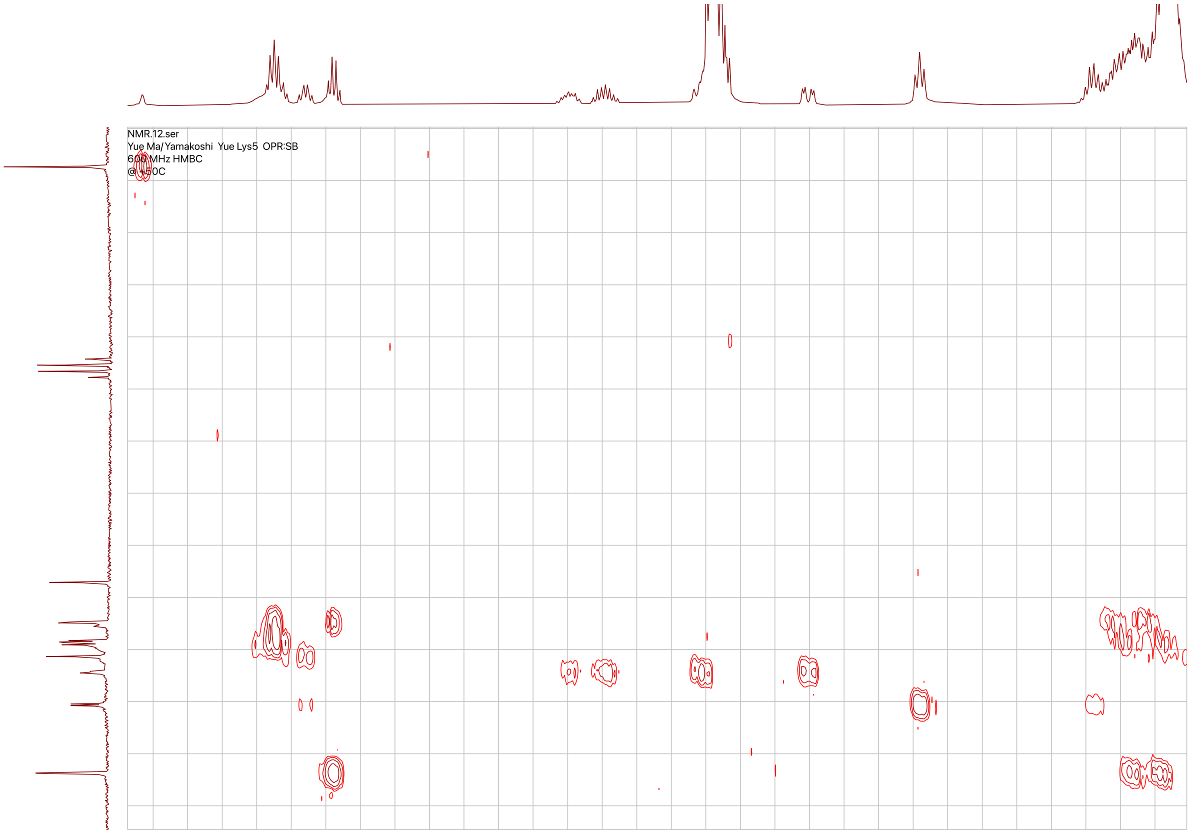

Figure S8. Expanded HMBC spectrum of **5a** in D_2O.

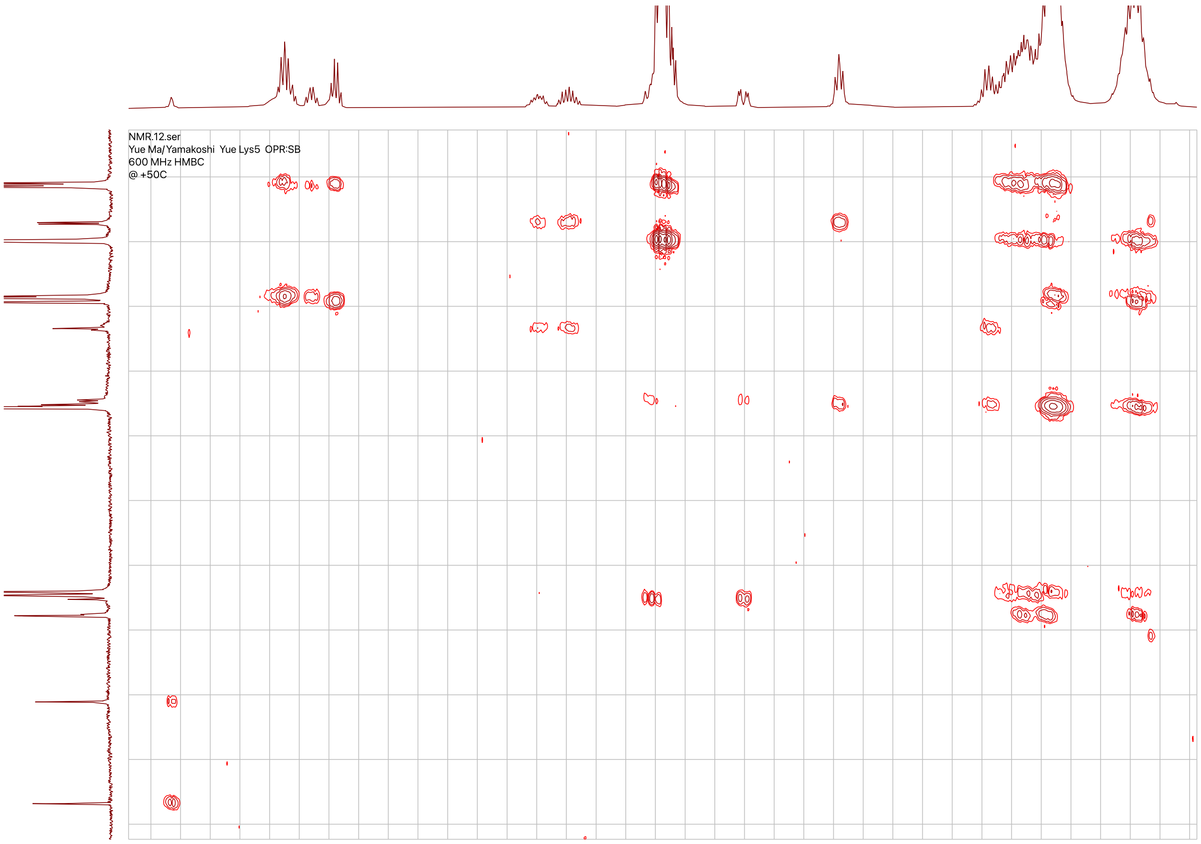

Figure S9. Expanded HMBC spectrum of **5a** in D_2O.

Scheme S2. Synthesis of C_{60}–oligo-Glu (**5b**).

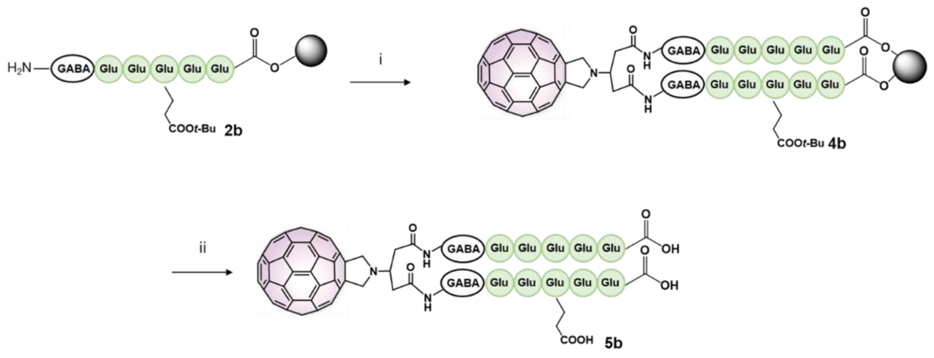

Reagents and conditions: i) **3**, HBTU, DIPEA, in DMF, rt, overnight, ii) TFA/TIPS/H_2O, rt, 1.5–2 h.

Peptide on resin 2b

The peptide was prepared on chlorotrityl resin (loading of 0.259 mmol·g^{-1}, 500 mg resin). The resin was subjected to the first addition Fmoc-Glu(O*t*-Bu)-OH. The automated peptide elongation was carried out on a

Biotage Syro I peptide synthesizer according to the general SPPS methods. The subsequent reactions were carried out with the following Fmoc-protected amino acids (4 equiv): Fmoc-Glu(O*t*-Bu)-OH, Fmoc-Glu(O*t*-Bu)-OH, Fmoc-Glu(O*t*-Bu)-OH, Fmoc-Glu(O*t*-Bu)-OH, and Fmoc-GABA-OH. Each coupling on the resin was carried out in presence of HCTU (4 equiv) and NMM (8 equiv) in DMF. Fmoc deprotection of each step was conducted by the repeated treatment of Fmoc-protected peptide on resin with 20% piperidine in DMF (two times for 10 min each). After each coupling reaction, the resin was washed with DMF. Fmoc-GABA-OH was coupled manually in the presence of 4 equiv of Fmoc-GABA-OH, 4 equiv of HCTU, and 8 equiv of NMM and deprotected by 20% piperidine in DMF to provide peptide on resin **2b**.

C_{60}–peptide on resin 4b

To the peptide on resin **2b** with a deprotected terminal amine (NH_2-GABA-EEEEE-resin), fullerene monoadduct **3** (13.3 mg, 0.015 mmol) in DMF (3.5 mL), HBTU (58.8 mg, 0.155 mmol), and DIPEA (37 μL, 0.335 mmol) were added. The mixture was agitated overnight, filtered, and washed with DMF and CH_2Cl_2 5 times to provide C_{60}–peptide on resin **4b**.

C_{60}−oligo-Glu (5b)

C_{60}–oligo-Glu with protective groups on resin **4b** was treated with a mixture of TFA/TIPS/H_2O (95:2.5:2.5, v/v) for 1.5–2 h, and the resin was removed by filtration. The filtrate was concentrated in vacuo, triturated with Et_2O, and centrifuged to obtain the crude peptide. The crude peptide was dissolved in TRIS buffer at pH 8.3 (25 mM) and subjected to spin filtration (three times for 3 h, 1.0 G, Millipore, Amicon Ultra-4, PLBC Ultracel-PL membrane, 3 kDa) to provide a light brown solid of **5b** (12.6 mg, 5.2 μmol, yield = 36%).

^{1}H NMR (600 MHz, 2% NaOD containing D_2O): δ 1.82–1.89 (m, 20H, Glu side chain C<u>H</u>$_2$CH$_2$COOH), 1.89–2.02 (m, 20H, Lys side chain CH$_2$CH$_2$C<u>H</u>$_2$CH$_2$NH$_2$), 1.90-1.96 (m, 4H, GABA NHCH$_2$C<u>H</u>$_2$CH$_2$CO), 2.08-2.26 (m, 20H, Glu side chain CH$_2$C<u>H</u>$_2$COOH), 2.84 (s, 4H, Prato adduct C<u>H</u>$_2$NH<u>C</u>H$_2$), 3.08-3.32 (m, 10H, Lys COC<u>H</u>(sidechain)NH), 3.32-3.39 (m, 4H GABA NHCH$_2$CH$_2$C<u>H</u>$_2$CO), 3.52-3.57 (m, 2H Prato adduct

$C\underline{H}_2CH(NH)CH_2$), 3.79-3.90 (m, 2H Prato adduct $C\underline{H}_2CH(NH)CH_2$), 4.07-4.12 (m, 2H GABA $NHC\underline{H}_2CH_2CH_2CO$), 4.21-4.39 (m, 1H, Prato adduct $CH_2C\underline{H}(NH)CH_2$); ^{13}C NMR (150 MHz, D_2O): δ 26.21 (GABA $NHCH_2\underline{C}H_2CH_2CO$), 27.89-28.87 (Glu side chain $\underline{C}H_2CH_2COOH$), 31.97-32.04 (Glu side chain $CH_2\underline{C}H_2COOH$), 32.25 (Prato adduct $\underline{C}H_2CH(NH)\underline{C}H_2$), 33.51 (GABA $NHCH_2CH_2\underline{C}H_2CO$), 40.13 (GABA $NH\underline{C}H_2CH_2CH_2CO$), 53.56-55.37 ($CO\underline{C}H$(sidechain)NH), 55.76 (1C, Prato adduct $CH_2\underline{C}H(NH)CH_2$), 63.82 (2C, Prato adduct $\underline{C}H_2NH\underline{C}H_2$), 70.91 ($sp^3$ $\underline{C}CH_2N$), 136-157 (sp2 cage region: 136.98 (4C), 140.67 (4C), 142.41 (4C), 142.43 (4C), 142.63 (4C), 142.91 (4C), 143.11 (2C), 143.62 (4C), 145.17 (4C), 145.81 (4C), 146.02 (2C), 146.58 (4C), 146.82 (4C), 146.97 (4C), 147.83 (2C), 156.05 (4C)), 172.82-176.35, ($\underline{C}ONH$); HRMS ($MALDI^+$) *m/z* calcd for $[C_{125}H_{96}N_{13}O_{36}]^+$: 2354.6075, found: 2355.6008 ($[M+H]^+$).

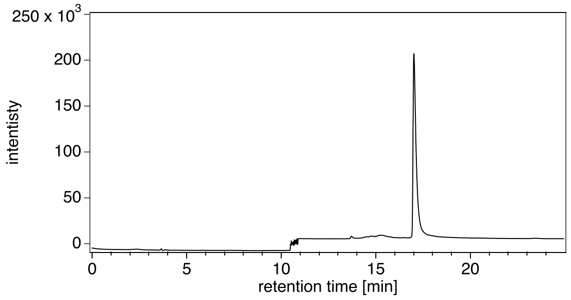

Figure S10. Analytical HPLC diagram of purified **5b** (column: Shiseido Capcell Pak C18 (20 mm × 250 mm), eluent: an isocratic system of CH_3CN/H_2O (5:95) for 3 min, a gradient system of CH_3CN/H_2O (5:95 to 95:5) over 14 min, an isocratic system of CH_3CN/H_2O (95:5) for 5 min, then an isocratic system of CH_3CN/H_2O (5:95), all in the presence of 0.1% TFA, flow rate: 1 $mL\cdot min^{-1}$, detection: 365 nm).

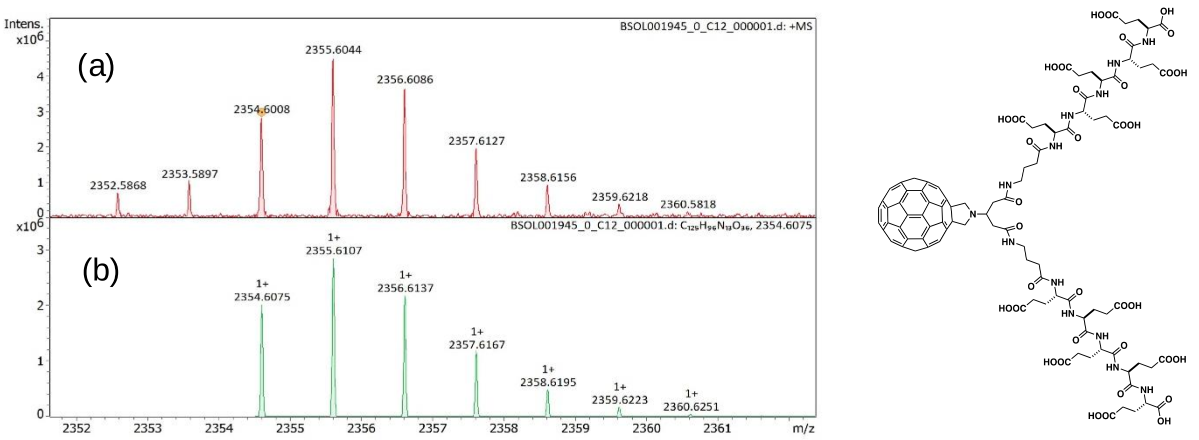

Figure S11. HRMS–MALDI spectrum of **5b**, measured (a) and simulated (b), and chemical structure.

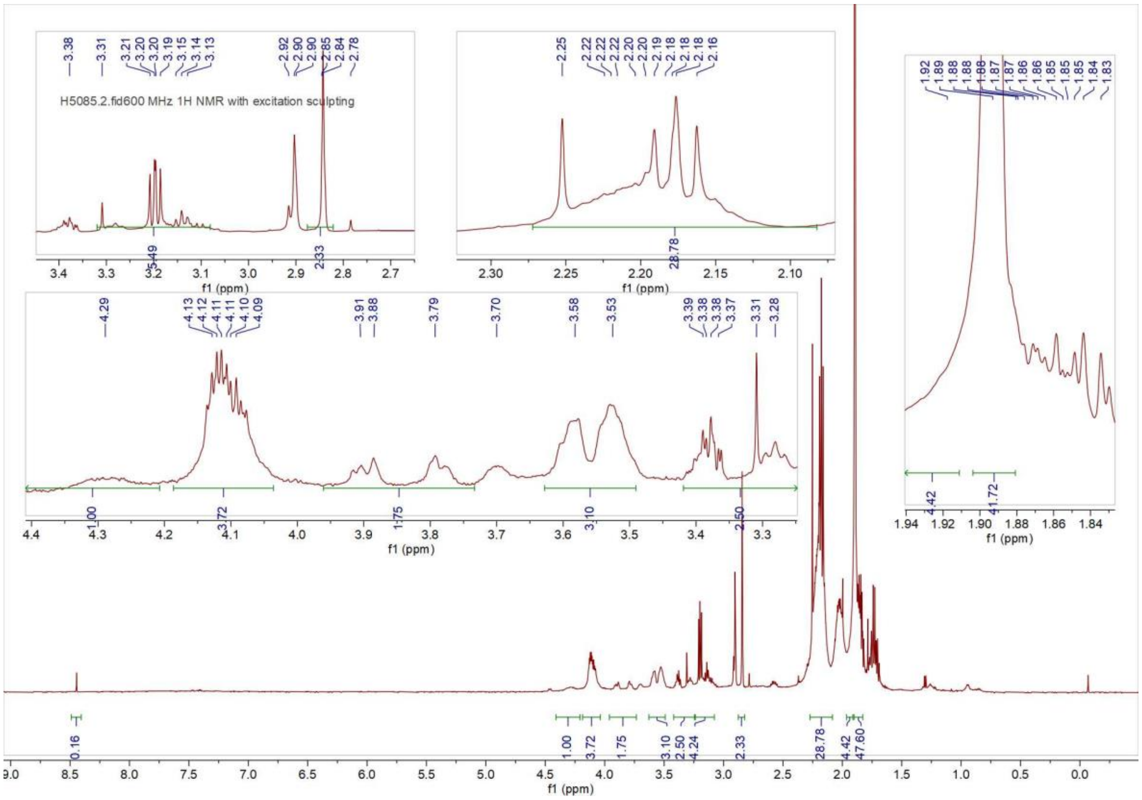

Figure S12. ^{1}H NMR spectrum of purified **5b** in D_2O with 2% NaOD (600 MHz).

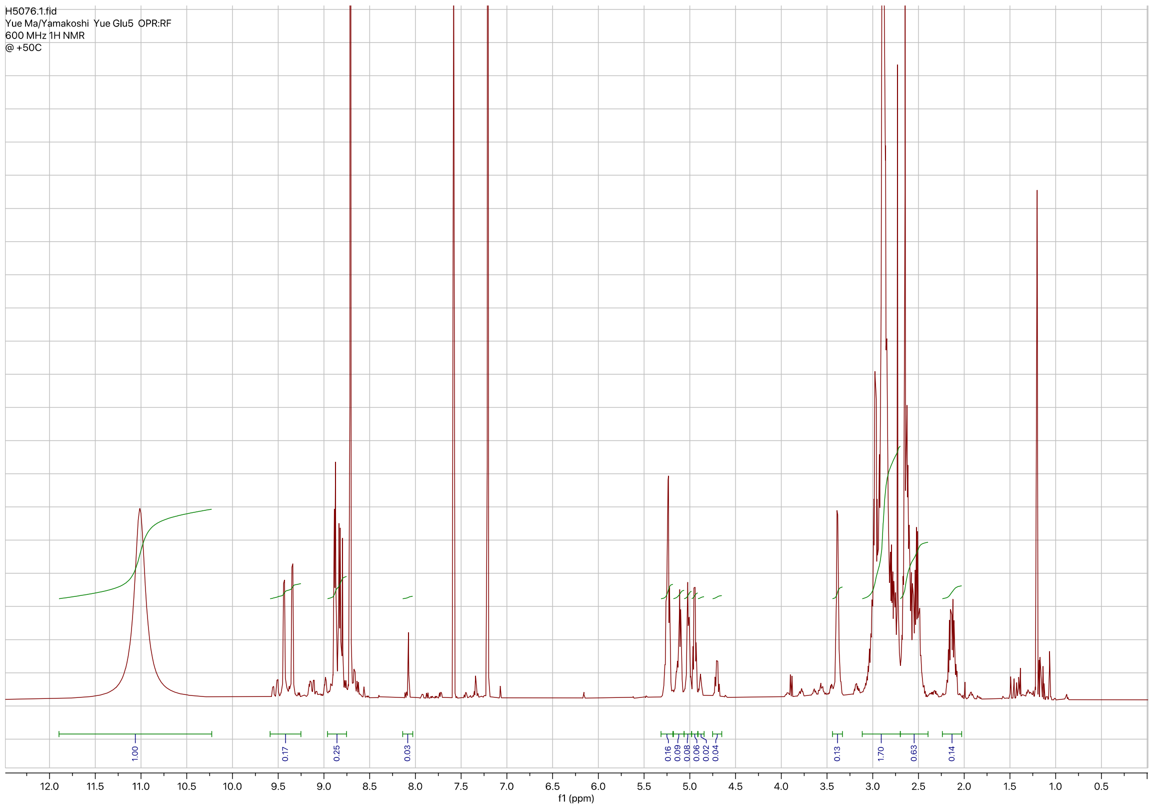

Figure S13. ^{1}H NMR spectrum of **5b** in pyridine-d_5 (600 MHz). The sample contains an impurity of oligo-Glu.

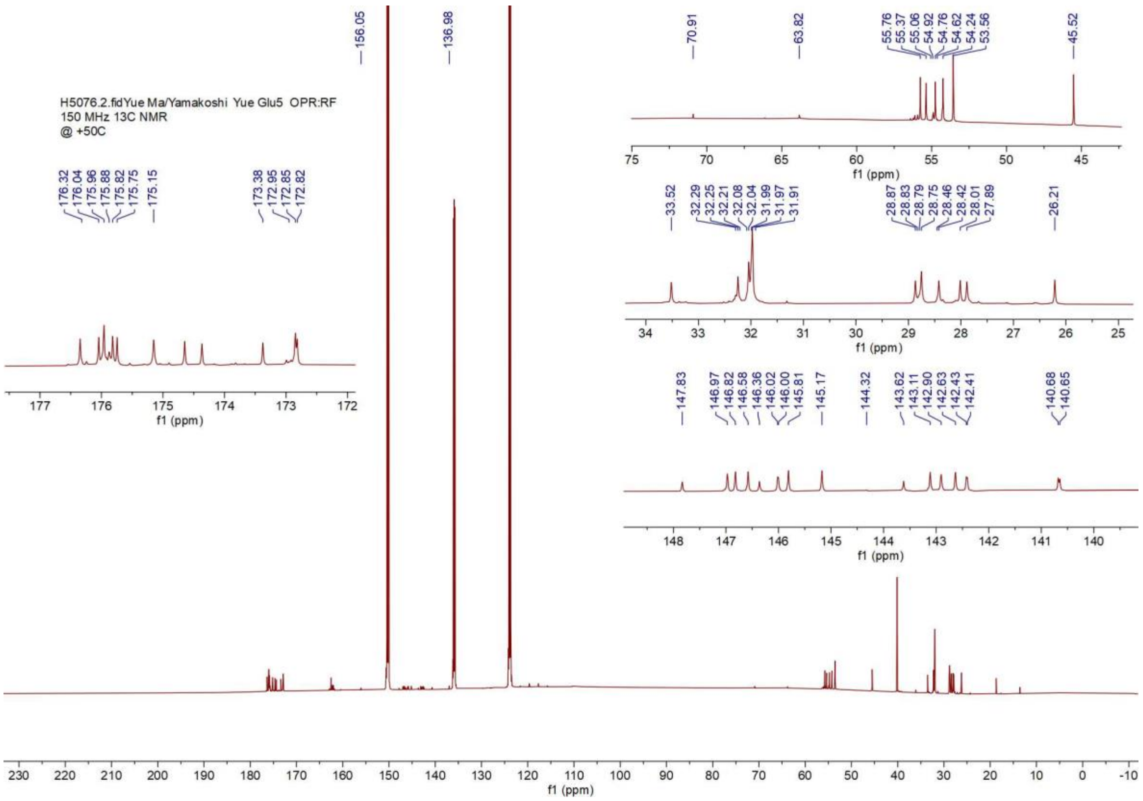

Figure S14. ^{13}C NMR spectrum of **5b** in pyridine-d_5 (150 MHz). The sample contains an impurity of oligo-Glu.

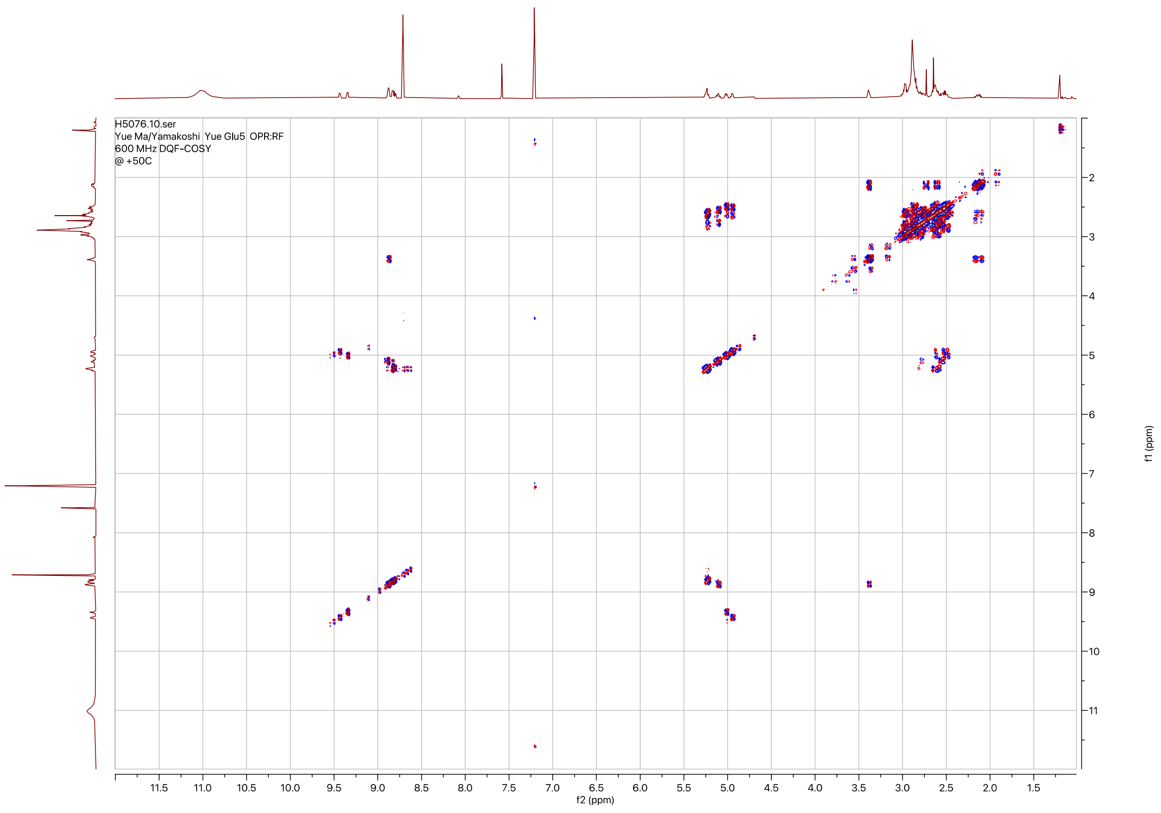

Figure S15. ^{1}H,^{1}H-COSY spectrum of **5b** in pyridine-d_5. The sample contains an impurity of oligo-Glu.

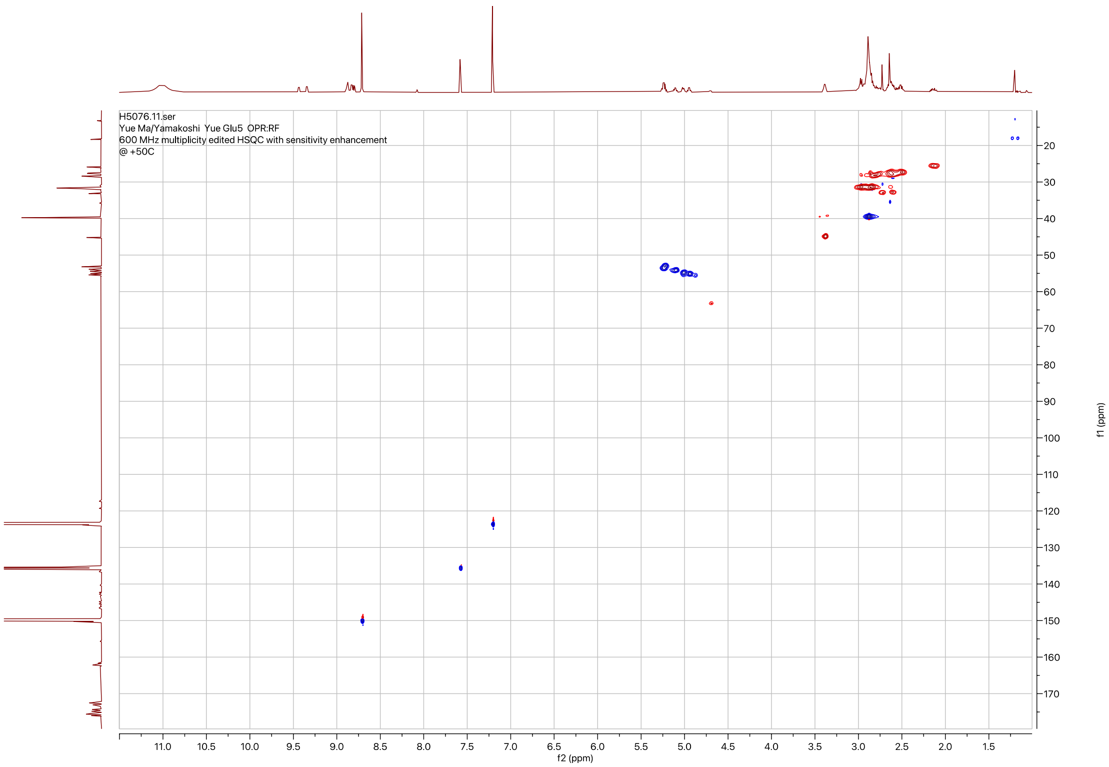

Figure S16. HSQC spectrum of **5b** in pyridine-d_5. The sample contains an impurity of oligo-Glu.

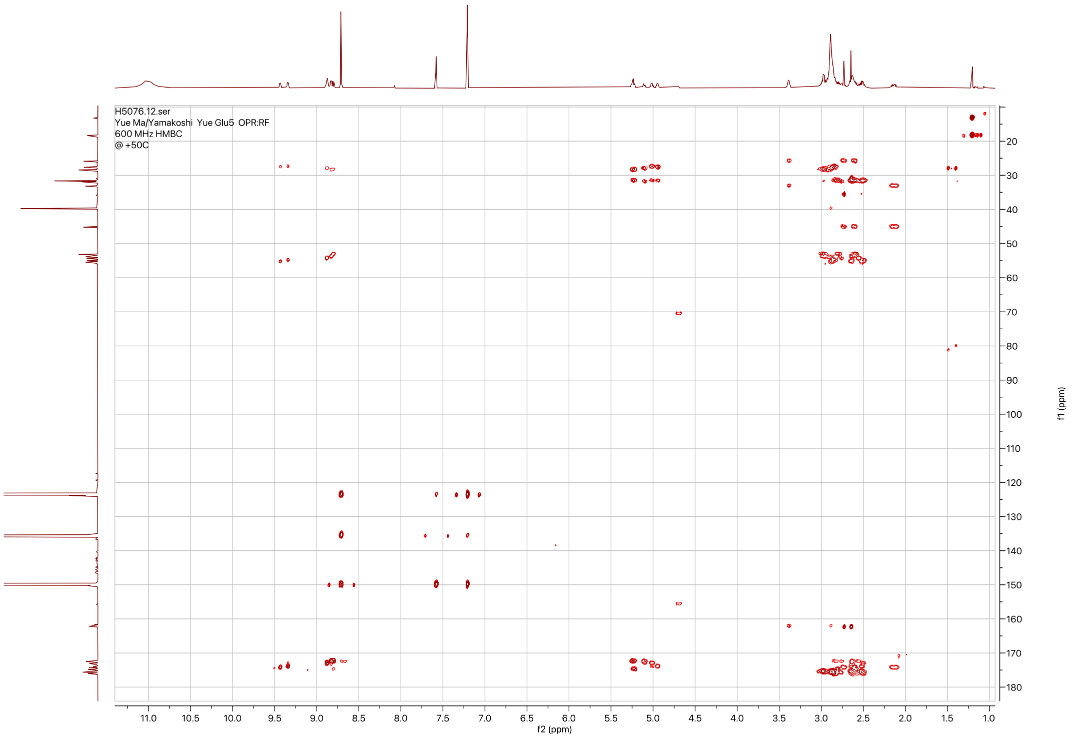

Figure S17. HMBC spectrum of **5b** in pyridine-d_5. The sample contains an impurity of oligo-Glu.

Scheme S3. Synthesis of C_{60}–oligo-Arg (**5c**).

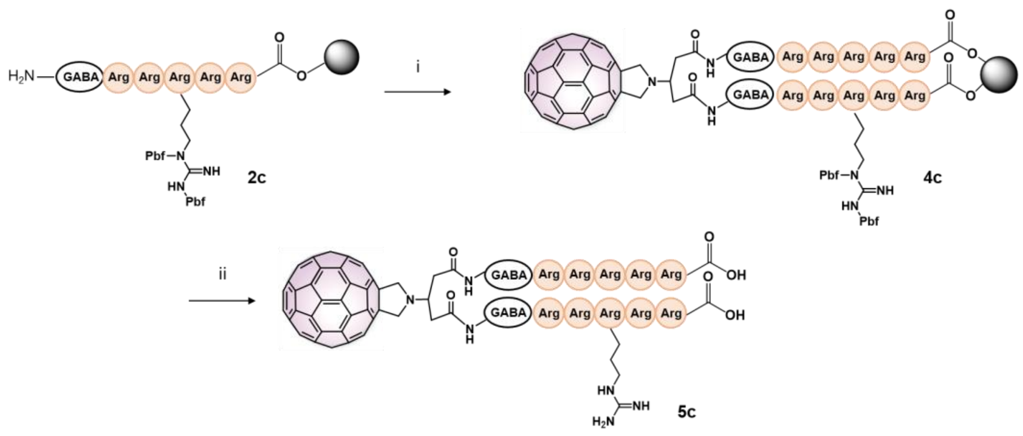

Reagents and conditions: i) **3**, HBTU, DIPEA, in DMF, rt, overnight, ii) TFA/TIPS/H_2O, rt, 1.5–2 h.

Peptide on resin 2c

The peptide was prepared on chlorotrityl resin (loading of 0.203 mmol·g^{-1}, 500 mg resin). The resin was subjected to the first addition Fmoc-Arg(Pbf)-OH. The automated peptide elongation was carried out on a

Biotage Syro I peptide synthesizer according to the general SPPS methods. The subsequent reactions were carried out with the following Fmoc-protected amino acids (4 equiv): Fmoc-Arg(Pbf)-OH, Fmoc-Arg(Pbf)-OH, Fmoc-Arg(Pbf)-OH, Fmoc-Arg(Pbf)-OH, and Fmoc-GABA-OH. Each coupling on the resin was carried out in the presence of HCTU (4 equiv) and NMM (8 equiv) in DMF. Fmoc deprotection of each step was conducted by the repeated treatment of Fmoc-protected peptide on resin with 20% piperidine in DMF (two times for 10 min each). After each coupling reaction, the resin was washed with DMF. Fmoc-GABA-OH was coupled manually in the presence of 4 equiv of Fmoc-GABA-OH, 4 equiv of HCTU, and 8 equiv of NMM and deprotected by 20% piperidine in DMF to provide peptide on resin **2c**.

C_{60}–peptide on resin 4c

To the peptide on resin **2c** with a deprotected terminal amine (NH_2-GABA-RRRRR-resin), fullerene monoadduct **3** (13.3 mg, 0.015 mmol) in DMF (3.5 mL), HBTU (58.8 mg, 0.155 mmol), and DIPEA (37 μL, 0.335 mmol) were added. The mixture was agitated overnight, filtered, and washed with DMF and CH_2Cl_2 5 times to provide the C_{60}–peptide on resin **4c**.

C_{60}–oligo-Arg (4c)

C_{60}–oligo-Arg with protective groups on resin **4c** was treated with a mixture of TFA/TIPS/H_2O (95:2.5:2.5, v/v) for 1.5–2 h, and the resin was removed by filtration. The filtrate was concentrated in vacuo, triturated with Et_2O, and centrifuged to the obtain crude peptide. The crude peptide was insoluble in most solvents and was obtained as a light brown solid **5c** (crude 37.2 mg, 9.8 μmol, yield = 66%).

HRMS (ESI $^+$) *m/z* calcd for $[C_{135}H_{149}N_{43}O_{16}]^{4+}$: 657.0536, found: 657.0540 ($[M+4H]^{4+}$).

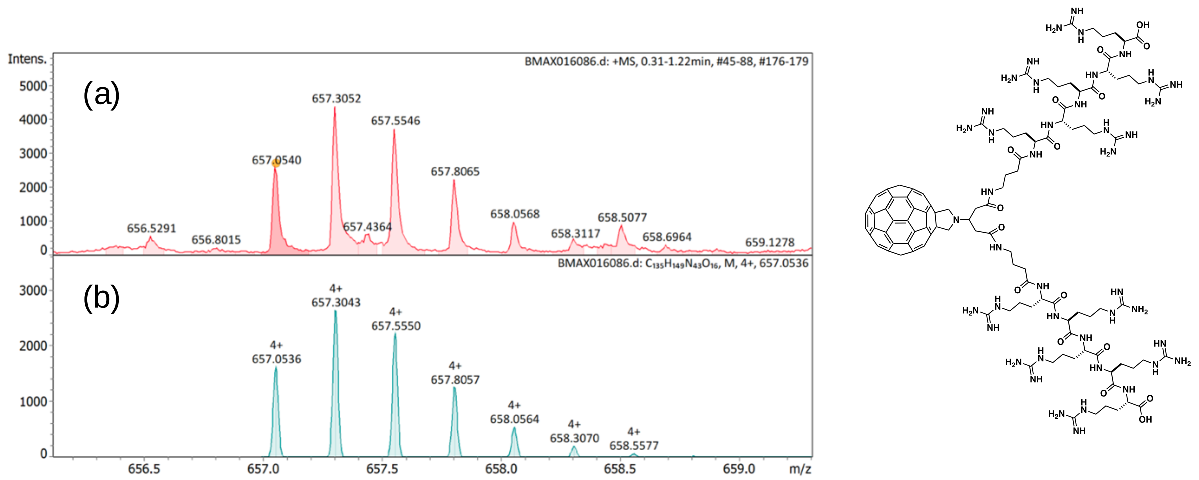

Figure S18. HRMS–ESI spectrum of **5c**, measured (a) and simulated (b), and chemical structure.

DLS

DLS measurements were performed on a Malvern Nano-ZetaSizer (Malvern Instruments Ltd., Worcestershire, UK), equipped with a 5 mW HeNe laser (wavelength: 632.8 nm) and a digital logarithmic correlator. C_{60}-oligo-Lys **5a** and C_{60}-oligo-Arg **5c** were measured with the concentration of 1 mM in miliQ water (pH=7.0). C_{60}-oligo-Glu **5b** was measured in with the concentration of 1 mM in miliQ water (pH=7.0) or in Tris buffer (pH9.0). All the measurements were performed at 25 ºC.

Solubility of C_{60}–oligopeptides 5a–c

Table S1. List of the solubilizing solvents for compounds **5a-5c**.

compound	solvent
C_{60}–oligo-Lys (**5a**)	water (pH 4.0–9.2), MeOH, DMSO
C_{60}–oligo-Glu (**5b**)	25 mM TRIS buffer (pH > 8.3), 2% NaOD in D_2O (pH 13.7)
C_{60}–oligo-Arg (**5c**)	— (insoluble in most solvents)

Detection of photoinduced $^1O_2$ generation by ESR spin trapping

ESR measurements were carried out on a Bruker spectrometer equipped with a microwave bridge X-band ER. Photoirradiation was performed by a green LED (Lumitronix, PowerBar V3, true green, 527 nm, 93 lm·W^{-1}, Osram Oslon SSL 150, in total 160 lamps were assembled in a cylindrical manner). Individual samples were loaded and sealed in a glass capillary (50 µL micropipette, Blaubrand® intraMark), which was subsequently irradiated for the appropriate time and then placed inside a thin-wall precision quartz ESR tube with a diameter of 4 mm and a length of 250 mm (Wilmad). Double integration of ESR spectra was performed with the WiNEPR processing program.

$^1O_2$ generation was detected through the $^1O_2$ adduct of 4-oxo-TEMP generated in an aqueous solution of C_{60}–oligo-Lys (**5a**) and rose bengal as reference under irradiation by a green LED (527 nm). The aqueous solution of **5a** or rose Bengal (0.1 mM in Milli-Q® water, 40 µL) was mixed with 300 mM phosphate buffer (pH 7.0, 20 µL), Milli-Q® water (32 µL), and 1 M 4-oxo-TEMP (8 µL) in a 4-mL vial and then bubbled with oxygen for 45 s. An aliquot (35 µL) of the solution was then sealed inside a glass capillary and then immediately irradiated by a green LED. Subsequently, the capillary was placed into a ESR tube for the measurement.
